# Do duplication-inducing elements ‘cooperate’ with genes in evolutionary arms races? A case study on cereal crop pathogenesis

**DOI:** 10.1186/s12870-025-07328-6

**Published:** 2025-10-30

**Authors:** M. Timothy Rabanus-Wallace, Thomas Wicker, Mohammad Pourkheirandish, Nils Stein

**Affiliations:** 1 University of Melbourne, Parkville, Australia; 2 Leibniz Institute of Plant Genetics and Crop Plant Research (IPK), Gatersleben, Germany; 3 University of Zurich, Zurich, Switzerland; 4 Martin-Luther-Universität Halle-Wittenberg, Halle (Saale), Germany

**Keywords:** Crop improvement, Pathogen resistance genes, Co-evolution, Segmental duplication, Cereals, Barley (*Hordeum vulgare*)

## Abstract

**Background:**

Crop improvement depends on the human ability to harness naturally- or artificially-occurring gene variants. Genomic segmental duplication can create redundant gene copies that can more freely ‘explore’ the space of possible mutations without adverse selective consequences. Such efficient generation of genetic diversity can be especially beneficial for organisms involved in evolutionary arms races such as the conflict between pathogens and their hosts. Given that some genomic regions are more prone to spontaneously duplicate themselves than others, we hypothesised that lineages in which arms-race-implicated genes fall within duplication-prone regions might enjoy a selective advantage, resulting in a measurable statistical association between the two.

**Results:**

We subjected the exceptionally repetitive and high-quality barley (*Hordeum vulgare* L.) genome assembly to a novel analysis to explicitly test, and confirm, that natural selection must have favoured lineages in which arms-race genes—in particular pathogen defence genes—are associated with duplication-inducers, most notably Kb-scale tandem repeats. Moreover, many well-studied pathogen resistance gene families such as NBS-LRRs and RLKs are independently identifiable by their associations with self-duplicating DNA. Such duplication-prone regions show a history of repeated long-distance ‘dispersal’ to distant genomic sites, followed by local expansion by tandem duplication. Often, the long tandemly duplicated motif differs between sites suggesting they arise often.

**Conclusions:**

The data support the view that genes in arms races have sometimes formed effectively cooperative associations with duplication-inducing sequences, supporting the view that some tolerance of genome-expanding genetic elements can be an evolutionarily advantageous strategy at the lineage level. Heavily duplicated genes are therefore more likely to be involved in arms races (such as pathogen defence) and hence may make suitable targets for crop improvement via targeted breeding or genome editing—as might the diversity-generating sequences they associate with.

**Supplementary Information:**

The online version contains supplementary material available at 10.1186/s12870-025-07328-6.

## Introduction

Genetic diversity is a critical resource for crop improvement. Humans have long exploited the diversity present in natural populations, discovering and combining beneficial genes and gene variants, and this process has been greatly accelerated by modern genomic technologies such as genetic markers, gene discovery, marker-based breeding, and genebank genomics. The directed de novo creation of commercially-useful genetic diversity via mutagenesis or genome editing has played a lesser role but proven its value with numerous successes. Mutagenesis in particular mimics the process of random mutation that produces the variation seized upon by natural selection in natural populations. Further exploitation of ‘natural’ mutational processes for crop improvement may arise as we increase our understanding and appreciation of the key mechanisms by which genes of relevance to agriculture evolve.

Segmental duplication is an important natural generator of novel genetic diversity [1]. A range of mechanisms have been characterised including non-homologous end joining, non-allelic homologous recombination (NAHR), strand slippage (SS) during replication, transposon-mediated copying, polyploidisation, and whole-genome duplication, as are moderating influences such as chromatin condensation state, recombination frequency, and other genetic and environmental factors [2–4]. Processes that reverse the genome-expanding effects of duplication by deleting segments are also well understood, leading to birth–death genome dynamics whereby genomic regions undergo continual recycling via recurrent expansion and degradation over long time scales [5, 6].

While the fate of most duplicated DNA is eventual decay, the potential benefits of segmental duplications—especially those that capture and multiply gene sequences—were predicted and discussed as early as the late 1900 s [1]. Phenomena such as neofunctionalisation of redundant gene copies [7], dosage modulation, fixed heterosis, and gene regulatory impacts have since been borne out by plentiful studies characterising particular instances, including many in plants that create phenotypes favourable for agriculture [7–11], especially as regards expanded families of genes associated with pathogen resistance [12–19].

The processes that cause DNA duplication, including tandem duplication and translocation, are dependent on certain existent DNA sequences. Local stretches of homology induce tandem duplications as a result of NAHR and SS. Transposable elements similarly increase the rate at which transpositions occur, some through considerably sophisticated means [3]. Duplication itself increases local homology, such that runaway expansion of repeats and even nested repeat expansion can occur [20].

Such observations led scientists as early as the 1970s to apply an analogue of the gene-centric view of evolution to duplication-inducing sequences, whereby the duplication-inducing unit in a genome is treated as a selfish replicator analogous to an organism in a natural environment. Such discussions have focussed on transposable elements in particular, but in the present study we consider a duplication-inducing element to include any DNA sequence that promotes the paralogous copying of any other nearby DNA sequence. Contrary to some contemporary conceptions [21], the selfish DNA paradigm discussed most actively during the 1980 s did not conclude simply that selfish- and junk DNA were synonymous [6, 22–26]. Rather, the literature recognised that genomic parameters such as the prevalence and multiplication rates of self-copying DNA, the copy numbers of genes, and even the genome size itself depended on interplay between multiple levels of selection, from the intra-genomic to the levels of organisms and populations.

The selective effects of duplication-inducing DNA at the organism and populations levels is expected to vary based on the configuration of selfish elements and genes, with more favourable configurations being increased in frequency at the population level over time. In the genomics era, studies on physically clustered pathogen-associated gene families recognise—explicitly or implicitly—that natural selection has favoured the results of gene duplication [12–19]. This is particularly relevant in the case of genes involved in arms races, that is, in antagonistic co-evolutionary conflicts, such as between genes for pathogen recognition and defence, and the corresponding genes in the pathogen that evade or suppress host immunity. Since genes in both the pathogen and host are forced to continuously adapt to the other’s evolving strategies, the multiplication (or ongoing birth–death ‘recycling’) of such genes by duplication-inducing elements may prove beneficial as it produces more opportunities to explore the space of possible beneficial mutations [1, 9, 27].

**Fig. 1:**
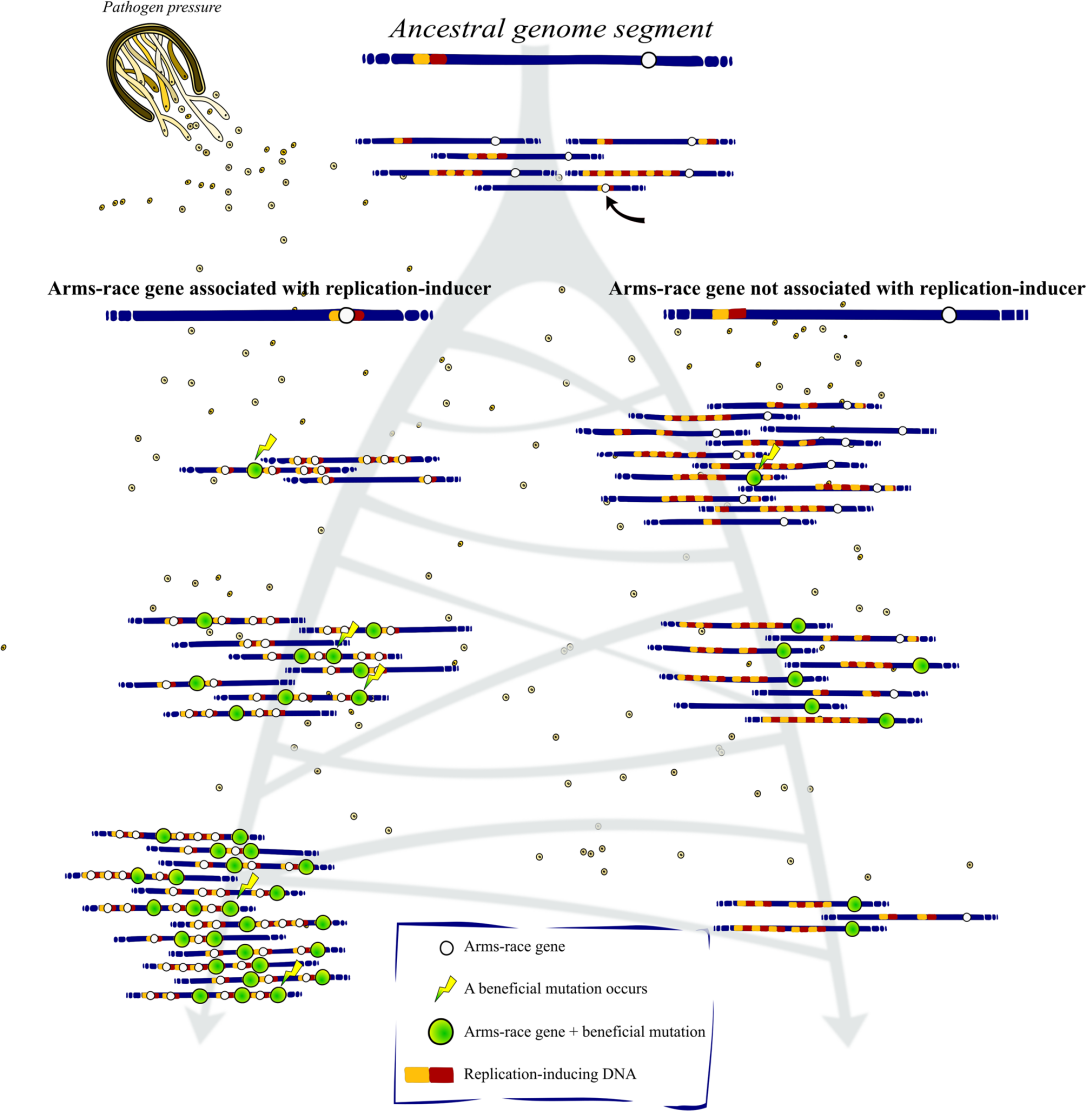
Selection favouring occasional novel variants generated by duplication-inducing elements will lead to measurable association between genes in arms races and duplication-prone genomic regions; a conceptual diagram. Navy strips represent the genomes of individuals in populations over time (grey arrows). A lineage arises in which a duplication-inducer + arms-race gene unit is established at low frequency in the population. Over time, the number of gene copies is amplified within this lineage. As a result, a greater number of mutations conferring a fitness boost are discovered, and natural selection increases the relative frequency of that lineage, leading to measurable gene—duplication-inducer association. The fungus and its spores here represent any kind of selection pressure with an arms-race dynamic (the species depicted is stem rust, *Puccinia graminis* Pers., a common barley pathogen). The sizes of repetitive regions are greatly exaggerated for clarity

Genome structural studies have clearly identified the long tandem repeats characteristic of NAHR- and SS-mediated gene multiplication in these cases [20]. Such discoveries speak to a more general hypothesis [25], specifically, that lineages in which duplication-inducing DNA elements act as effective diversity generators for genes involved in evolutionary arms-races enjoy an overall selective benefit [23]. In the language of selfish replicators, duplication-inducing elements effectively *cooperate* with genes in arms races (henceforth, *arms-race genes*), since both elements benefit from the association. Over generations, this process of selection favouring lineages in which arms-race genes are physically associated with duplication-prone genomic regions is expected lead to a measurable association between the two (Fig. 1).

**Fig. 2:**
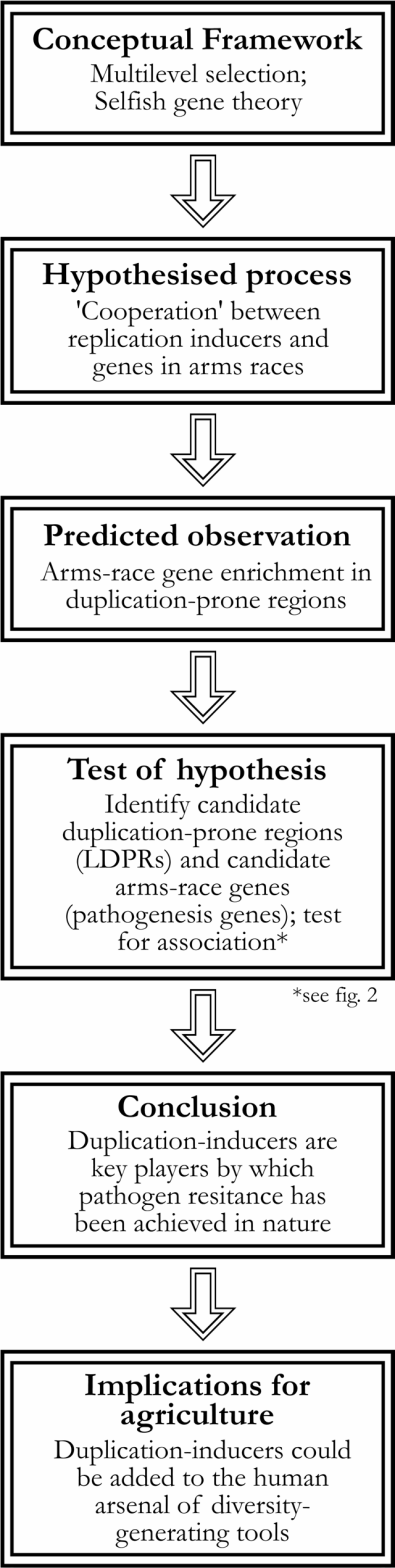
Outlining the logic of the study. For a discussion of how LDPRs are specifically defined see the Overview subsection under Results

The present study aims to bring the highly detailed data available from advanced genome assemblies to definitively prove this association exists, and on this basis to argue that wider adoption of the language of selfish replicators (especially of cooperation between genomic elements) might facilitate new approaches towards diversity generation for crop improvement, of which we suggest several concrete examples (Fig. 2). The Barley (*Hordeum vulgare* L) genome was selected as a study case owing to (i) its importance as a model agricultural cereal, (ii) its annual life cycle and pathogen susceptibility which guarantees the presence of genes in arms races, many of which have been extensively studied, and (iii) the availability of a contemporary, highly-accurate assembly of its exceptionally repetitive diploid genome.

## Results

### Overview

**Fig. 3:**
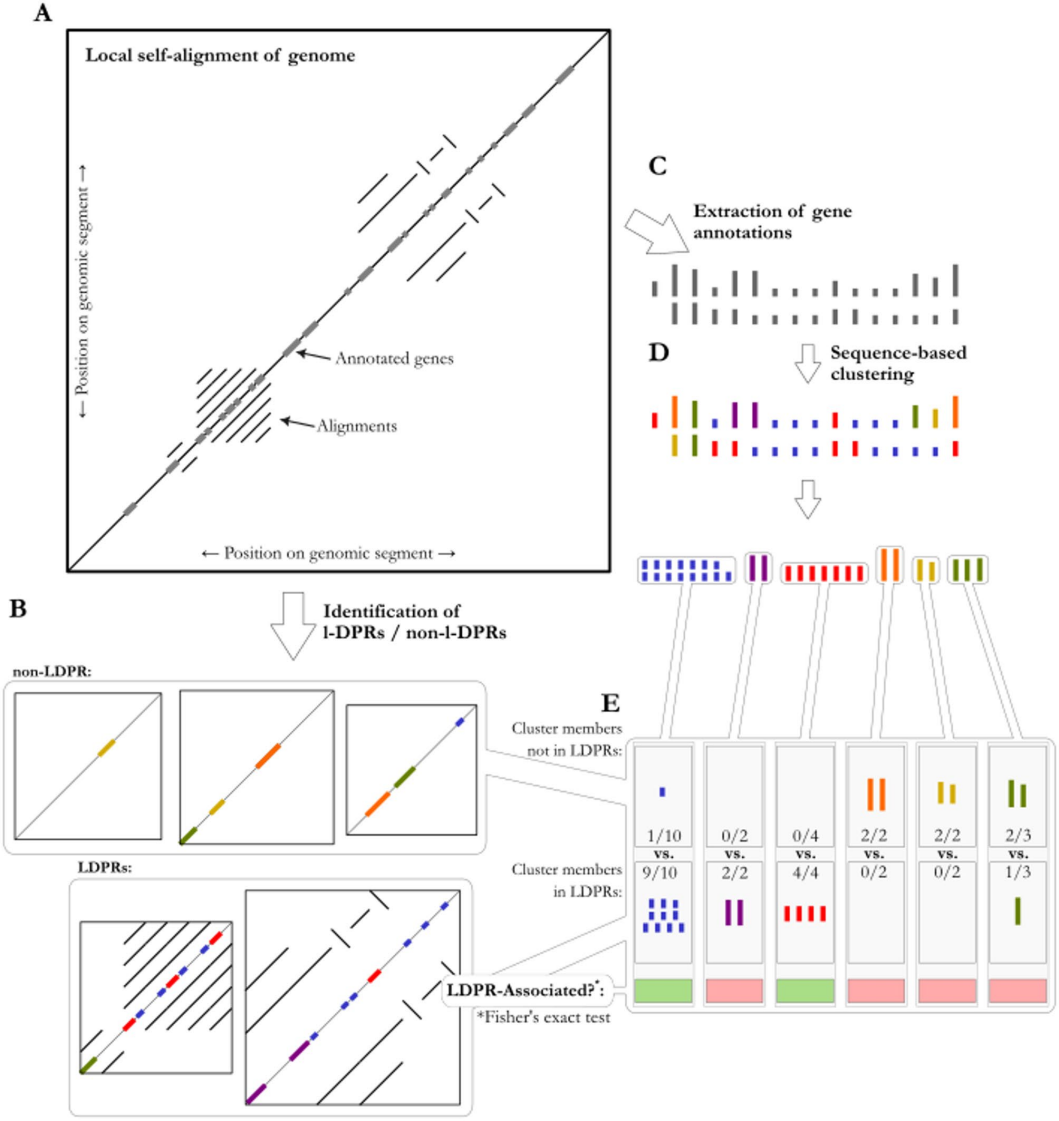
A method for testing the association of LDPRs with particular gene clusters. Local self-alignments of the genome assembly (**A**) were used as a basis for classifying genomic ranges as LDPR or otherwise (**B**). Genes from the MorexV3 annotation (**C**) were assigned to gene clusters based on their sequences (**D**), and the independence of gene cluster membership from LDPR association was tested for each gene cluster (**E**)

Our approach depended on developing a approach to tabulate a list of regions of the MorexV3 reference assembly of *H. vulgare* cv. ‘Morex’ [28] that show evidence of containing sequences that promote large local duplications. We name these Long-Duplication-Prone Regions (LDPRs) and operationally define them only as regions with elevated levels of duplicated sequences above a certain length (see methods for details), without focussing ab initio on the kinds of repeats (e.g. tandem, translocation, transposable element) that they are composed of. We then listed gene clusters that are statistically over-represented in these LDPRs (Fig. 3). Finally, we used gene functional descriptions from the MorexV3 annotation [28] to test whether this list of LDPR-associated gene clusters shows a better-than-chance amount of overlap with a list of previously identified candidate arms-race-associated gene clusters compiled from the literature. For the purposes of practicality, our list of arms-race candidates features mainly pathogenesis-related genes—which are well-studied in cereals—compiled based on the research literature and the GO terms/gene descriptive terms of the MorexV3 annotation (see methods). This is simply a matter of practicality, however, and we continue to use the term arms-race gene because the theory from which our hypothesis arises predicts the same should be true of any arms-race gene, not just those we are best positioned to identify.

### A gene-agnostic approach to identifying duplication-prone regions, and gene families that associate with them

**Fig. 4:**
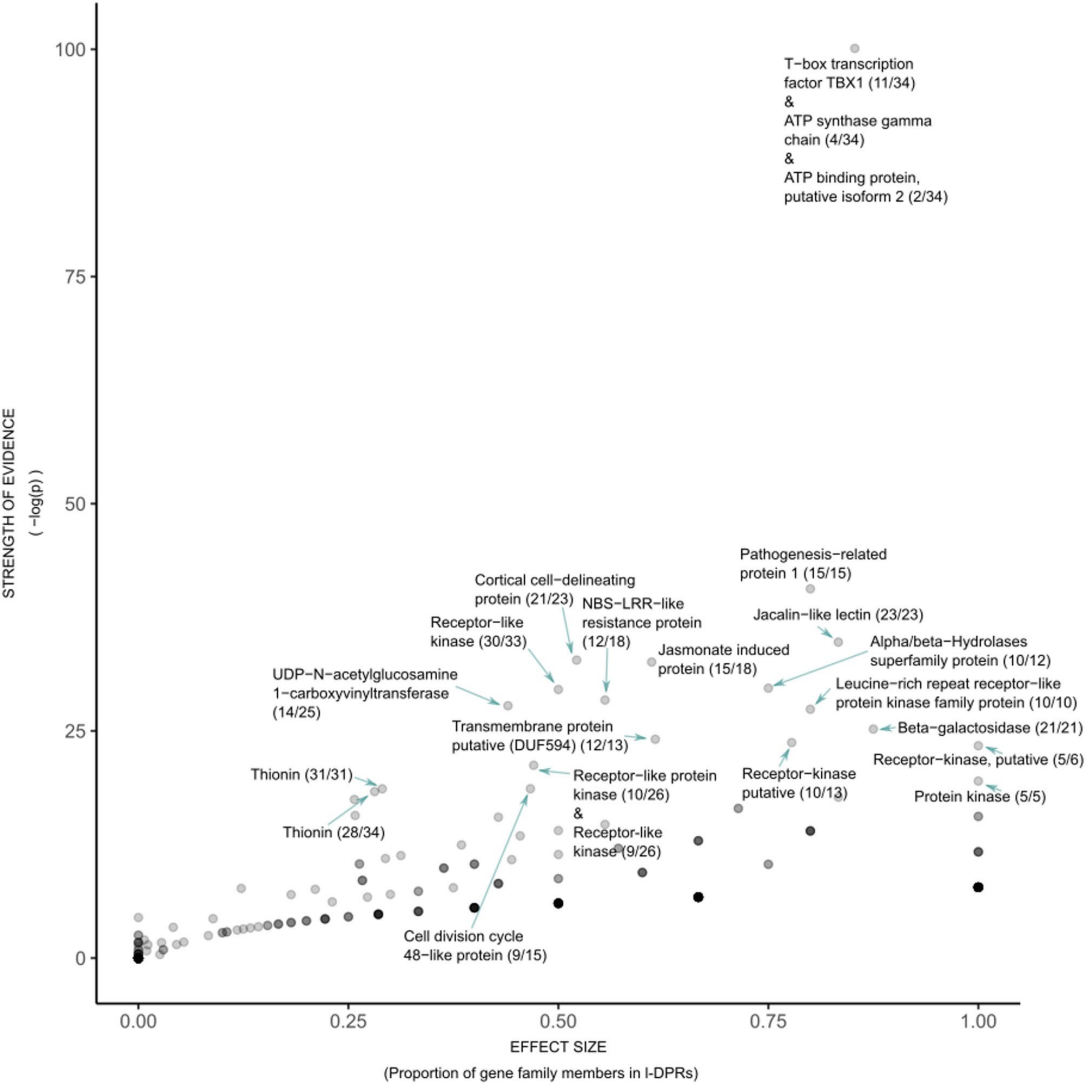
Volcano plot summarising gene cluster association with LDPRs, Gene clusters in the upper right part of the plot are significantly more frequently associated with LDPRs than they would be under random assignment (Y-axis), and the difference between their frequencies in- and out of LDPRs is larger (X-axis). Gene clusters with strong evidence of association with LDPRs are marked with their most-common homology-based descriptors as provided by the MorexV3 annotation. In parentheses: (number of descriptors in gene cluster sharing most common descriptor/total number of gene cluster members). Where the most common descriptor does not account for at least half the descriptors, further descriptors are listed. Note the majority of unlisted descriptors differ only trivially from the most common (capitalisation, hyphens, etc.). The strongest evidence of association is given to gene cluster cl_16606, a cluster of very short proteins with mixed homology-based descriptors such as “T-box transcription factor” (a gene family found only in animals). Unlike other gene clusters discussed in this paper, these very short predicted proteins without strong evidence of expression are likely pseudogenes or annotation errors (see Supplementary Notes 1)

A novel approach was conceived to identify LDPRs based on scanning genome self-alignments for intervals with an elevated amount of locally-repeated sequences in the Kbp-scale length range (see methods). Our LDPR discovery pipeline identified 1,199 candidate LDPRs (Supplementary Table 1) with lengths ranging between 5.5 and 1,123.598 Kbp (median length 33.600 Kbp), located primarily in the subtelomeric regions of all seven chromosomes (Supplementary Figures S2). Annotated genes given a “high confidence” (HC) ranking by the MorexV3 annotation pipeline [28] were assigned to 17,186 clusters based on protein sequence similarity, comprising between 1 and 727 members each (Supplementary Tables 2—3), most of which (67.2%) were singleton clusters, and 458 of which qualified as members of the arms race pool. Even before explicitly classifying genes or testing for associations, inspection of the orthology-derived descriptor terms for LDPR-associated gene clusters revealed many terms relating to known pathogen-related functions in barley and other cereals, including within the top ten terms (Fig. 4; Supplementary Tables 4) ‘pathogenesis-related protein 1′ [29], ‘jacalin-like lectin’ [30], ‘receptor-like kinase’ [31],’jasmonate-induced protein’ [32], ‘thionin’ and ‘thionin-like peptide’ [19, 33], and ‘leucine-rich repeat’ [12]. Also present among the gene clusters’ common descriptors are `alpha/beta-hydrolase superfamily` [18], whose members have broad but poorly-characterised functions in plants which do include hormone reception, and `Cortical cell-delineating protein` [34], also poorly-studied, which is known to express in the cortical ground meristem of maize roots, accumulating near the region of fastest cellular elongation.

### Duplication-rich regions are enriched in arms-race genes, and include many known pathogen interaction genes

We went on to statistically confirm the association of arms-race genes with LDPRs. Gene clusters associated with LDPRs were significantly more likely to be arms-race genes (likelihood ratio test, *p* = 0.00239; Supplementary Table 5), implicating function-based selection as a force shaping the genic composition of duplication-prone genomic regions.

### The analysis method produces the expected results when tested on housekeeping genes

To confirm the results of the analysis method described thus far are robust, we repeated the entire pipeline substituting our pool of candidate arms-race genes, for something like its opposite—a pool of genes highly unlikely to be involved in arms races. For this purpose we defined our test set of 996 genes by close homology to a list of barley housekeeping genes, compiled as expression standards by Gines et al. (2018) [35]. As expected, they produced no evidence of association between housekeeping gene clusters and LDPR-associated clusters (likelihood ratio test, *p* = 0.2953; Supplementary Table 5).

### The genomic structure surrounding candidate RGSUs suggest duplication-inducers display migrate-and-expand dynamics

**Fig. 5:**
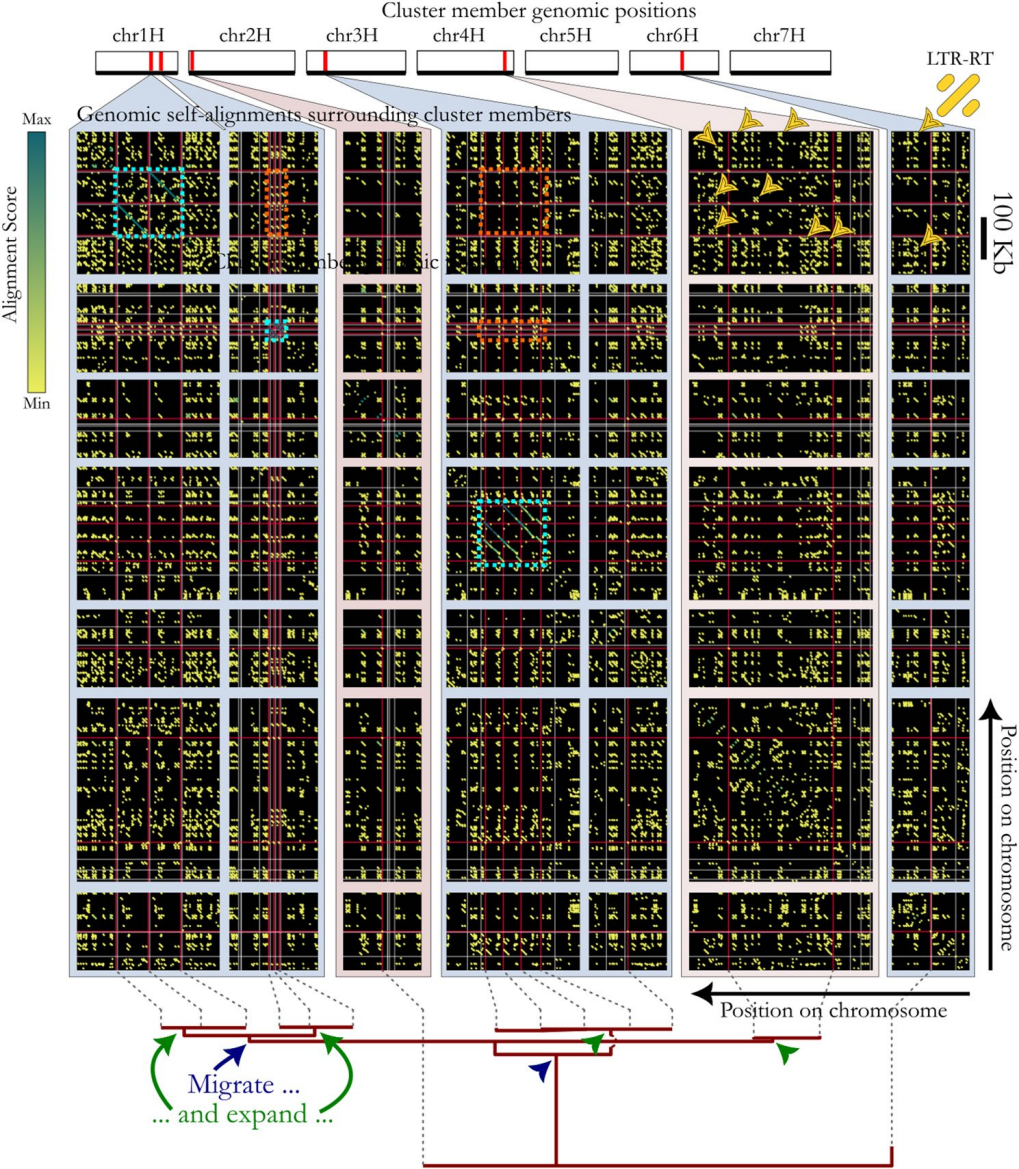
Evolutionary dynamics of a group of related LDPRs containing arms-race-associated gene cl_1888. Top: Positions of gene cluster members on barley chromosomes 1H—7H; Middle: Alignment plots at the LDPR scale, showing alignments longer than 50 bp surrounding the gene cluster members. Alignments between paralogous segments are indicated by lines with the LASTZ alignment scores presented as colours (scaled so max → min corresponds to dark blue → yellow). The positions of genes that are members of cl_1888 are indicated with red bars, white bars indicate genes from other gene clusters. Since the plot is symmetrical around the diagonal, the gene positions are shown on both the horizontal and vertical axes. Blue rectangles indicate where long tandem duplications have created new gene copies, and orange rectangles show that these long tandem duplications are not conserved between different amplification sites. Yellow arrowheads are included to draw the reader’s attention to the canonical appearance of LTR-retrotransposons in alignment plots. Bottom: ML gene tree estimating the ancestral relationships between gene cluster cl_1888 genes based on protein alignment. Blue and green arrowhead respectively point to branches showing either ‘dispersal’ (long-distance transposition) or ‘expansion’ (local duplication) events

**Fig. 6:**
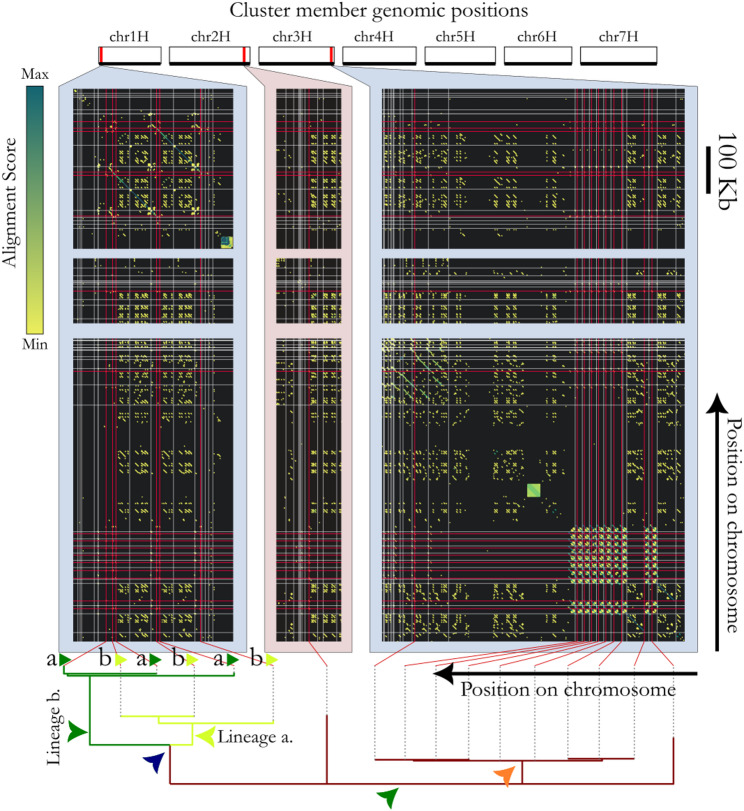
Related LDPRs, containing gene cluster B (cl_8856). Figure features follow Fig. 5. A case of temporally-separated tandem duplication events is implied by the separation of lineages a and b (labelled bottom left), which appear in alternating order, Suggesting Subsequent tandem duplications of the pair together. Note the very close similarity between members of the cluster on chromosome 3H (orange arrow), suggesting recent expansion (see also Supplementary Materials 2)

Examination of sequence alignment plots between regions containing LDPR-associated gene clusters (e.g., Figs. 5 and 6 showing clusters cl_1888 and cl_8856, Supplementary Figure S3 showing cluster cl_16606, and Supplementary Materials 1) suggest several common features. We frequently see long-distance dispersal followed by tandem duplication at the new landing site. Notably, however, the tandemly repeated units at each site are usually not similar. In the case of gene cluster cl_1888 (Fig. 5), (whose members are annotated as jasmonate-induced proteins, having roles in pathogen response signalling [28, 32]) protein phylogeny and alignments (Supplementary Materials 2) confirm cluster members have been locally duplicated as part of completely distinct tandemly-arrayed motifs—one on chromosome 1H, and two on chromosome 3H. The same pattern of distinct tandem repeat motifs on different chromosomes is evident for cluster cl_8856 (Fig. 6). Additionally in this cluster, a tandem repeat array on 1H displays alteration between two variants of the gene in an a-b-a-b-a-b arrangement, suggesting a single ancient tandem duplication event, followed by a pair of recent events that duplicated a larger segment containing the initial duplication to create a nested structure. Gene clusters cl_15653 (members annotated as thionin genes [28]), cl_14902 (annotated as pathogenesis-related proteins [28]), cl_15128 (annotated as cortical cell-delineating proteins [34]), and many others all evidence the same phenomenon (Supplementary Figures S3; Supplementary Materials 1). The discovery of these tandem-repeat-structured LDPRs on many different chromosomes therefore suggests they are associated with sequences that induce gene copying across long distances, and tandem duplication at new transposition landing sites—effectively a transpose-then-duplicate strategy at the genome level [36].

While we initially suspected LDPRs harbouring repeated genes to be made up of predominantly transposable elements, tandem duplicated arrays appear to be the dominant gene duplication mechanism (e.g. by examining Supplementary Materials 1). LDPR-associated gene cluster members that do not fall within a tandemly repeated region tend to be associated with dense local expansions of LTR retrotransposons (recognisable by their canonical “percent-sign” appearance in alignment plots, as shown in Fig. 5 depicting gene cluster cl_1888, whose members are annotated as cell division cycle 48-like proteins). For further examples, see cl_15128 and cl_1274 (annotated as NBS-LRR-like resistance proteins; Supplementary Materials 1).

Gene cluster cl_16606 is included as Supplementary Figure S3 to illustrate the possible utility specifically of tandem repeat induction as a means of creating gene redundancy *en masse*, for instance, in breeding programs (see Discussion). Close homology between spatially separated runs of repeat units clearly reveal the duplication of whole subarrays of various sizes. Despite a lack of evidence for genes in cl_16606 being an actively expressed (see Supplementary Notes 1), the LDPRs with which it associates make apt examples to describe the mechanism of LDPR proliferation; LDPRs containing cl_16606 are discernible on at least five different chromosomes. The tandemly-duplicated unit is almost certainly also the unit that induces translocation, since it is shared between all LDPRs involved. Furthermore, we can observe evidence that the duplication events can involve up to at least ~ 12 units at a time, resulting in increased runs of similarity between non-consecutive but equally-distant units owing to their recent common ancestry (yellow arrows in Supplementary Figures S3). This occurs most strikingly within the chromosome 1H LDPR shown in Fig. 6. In arrays on chromosome 5H and 6H, homology between neighbours increases in a graded manner towards one end of the array, suggesting unidirectional recent expansion.

### Activity of genes in gene clusters cl_1888, cl_8856, and cl_16606

There is ample evidence for active expression of gene clusters cl_1888 and cl_8856 (Figs. 5 and 6). Evidence regarding cluster cl_16606’s (Supplementary Figures S3) function and expression is inconclusive (Supplementary Notes 1 and Supplementary Table 6). While it is not necessary to the analysis that every LDPR-associated gene be functional, expression data clearly indicate the activity of several genes identified in such LDPRs.

## Discussion

### Mechanisms of proliferation

Examples such as gene clusters cl_1888, cl_8856, cl_16606, and others (see Results; Figs. 5 and 6; Supplementary Figures S3; Supplementary Materials 2) suggest that some gene clusters become associated with expanding tandemly repeating structures (i.e., a type of duplication inducer) at multiple genomic locations, but that the tandemly repeated motifs at different locations are not identical. This naturally invites the interpretation that ancestral gene copies were transposed to remote parts of the genome, following which they became involved in local duplications at each new location, possibly to the benefit of the host organism. Duplication inducers, it would appear, must arise frequently—and the widespread occurrence of LDPRs across the genome is consistent with this. Gene duplication by NAHR and SS occur when two ‘seeds’ of similar sequence occur nearby each other, and result in duplication of the entire segment between the seeds. This initial duplication effectively produces extra seeds for future potential duplication events. Previous studies have indicated that certain seed repeats of a few 100 bp in length and with ~ 90% sequence identity are sufficient as templates for NAHR [3], but in the case of the barley genome at least, our data suggest transposable elements (TEs) may play a key role acting as potential seeds for duplication. In barley and wheat, 50% of the genome is derived from only about 10 high-copy TE families [17, 37]. This degree of abundance means that there is a high probability that a given gene is flanked by TEs of the same family (and in the same orientation), providing suitable seeds to initiate regional duplication. This is consistent with our alignment plots (Figs. 5 and 6, Supplementary Figures S3, Supplementary Materials 2) providing abundant examples where fragmentary or whole LTR-retrotransposon signatures are present in the vicinity of and even within gene-containing tandem repeats. Studies have shown that while certain barley TE families inhabit the same subtelomeric genomic ‘niche’ as do the majority of LDPRs (Supplementary Figures S2), genes, and recombination events, the vast majority occur in the low-recombination subcentromeric zones [37, 38]—suggesting recombination may be a key factor in of the formation of LDPRs, i.e. that NAHR is more important than SS. Yet other studies have shown TEs play a role in transporting microsatellites around the genome [36].

### If seeds that cause tandem duplication are often beneficial, it could help explain the tolerance of TEs in genomes

This view bears on discussions about the ‘selfish’ versus ‘adaptive’ role of TEs in the genome, supporting the view that TEs themselves reproduce selfishly, but *tolerance* of TEs—a known heritable trait [5, 38–40]—is responsive to selection at the lineage level: Specifically, our findings align with the hypothesis that some tolerance of active TEs opens opportunities to creating adaptively beneficial genes, but logically there must be some upper limit of TE tolerance at which genome bloat becomes detrimental, leading to a (static or dynamic) equilibrium [5].

### Implications for agriculture

Co-evolutionary arms races are of great importance in modern plant agriculture. Elite domesticated plant varieties are genetically near-identical, and pathogen strains that evolve means to circumvent their defences can rapidly cause widespread crop failure with devastating human impact. Genetic interventions to improve pathogen resistance consist in identifying and transferring genes that confer resistance to known pathogens into high-yielding varieties, via breeding or genome editing. The identification of genes likely to benefit cultivated varieties by conferring broader pathogen resistance is equivalent to the problem natural selection solves when wild plant populations leverage a store of accumulated genetic diversity to overcome new pathogen strains, reflected by an extensive literature on the roles of duplicated genes in key crop domestication traits [8, 41]. We propose that, if it can be shown that known arms-race genes associate with duplication-inducing sequence, then it follows that all gene families associated with diversity-generating duplication hotspots should be considered candidate genes for increasing the resistance of cultivated crops to a broad range of co-evolving threats. The preponderance of known pathogenesis-related gene families identified in our study, based purely on their association with LDPRs is consistent with findings from the genomics literature surveying the effects of gene duplication in plants including barley [7, 8], and a strong quantitative indicator that the method may have predictive power to identify lesser-studied gene families with agricultural potential. Such gene families include those with descriptors ‘Cortical cell-delineating protein’ [34] (cl_15128) ‘Beta-galactosidase’ [42] (cluster cl_1797), ‘transmembrane protein, putative (DUF594)’ [43] (cl_1895), and other entries in Supplementary Table 4.

Moreover our study strongly reiterates the central importance of duplication-inducers as diversity generators, which based on (e.g.) the abundance of near-identical proteins in LDPR-associated gene clusters (Supplementary Materials 2), appear to act over very short evolutionary timespans. That these diversity factories have been seized upon by natural selection to fight wild pathogens may indicate a promising avenue in the use of artificial selection to fight pathogens of domesticated crops. By ensuring ample supply of LDPRs inhabit the future genetic pools drawn from in resistance breeding efforts, we may help maintain a diverse arsenal of potential resistance genes over cycles of artificial selection, and similarly, maintaining LDPRs in agricultural cultivars might even enable some beneficial unconscious selection of beneficial new variants in the course of seasonal planting and seed multiplication cycles.

## Methods

### Gene-agnostic identification of long-duplication-prone-regions (Supplementary Figure S1)

The pipeline achieves sequence-agnostic identification of LDPRs based on the assumption that a candidate LDPR will meet four criteria: It will contain (i) an elevated concentration of (ii) locally (iii) repeated sequences in the (iv) Kbp-scale length range. We first aligned the MorexV3 against itself using lastz [44] (v1.04.03; arguments: ‘–notransition –step = 500 –gapped –self’). Lastz is a dedicated genome-to-genome aligner, and was chosen for its algorithmic similarity to popular aligners such as BLAST in addition to convenient features such as the `step` parameter accelerating the search, ability to align Subsets of the Subject sequences without first extracting them into new files, and the dedicated self-alignment mode which, unlike BLAST, guarantees symmetry of alignments around the diagonal. For practicality purposes, self-alignment was done in 2 Mbp blocks with a 200 Kbp overlap, and any overlapping LDPRs identified in multiple windows were merged. For each window, we ignored the trivial end-to-end alignment, and of the remaining alignments, retained only those with a length exceeding 5 Kbp, and falling fully within 200 Kbp of one another. An alignment ‘density’ was calculated over the chromosome by calculating, at ‘interrogation points’ spaced equally at 1 Kb intervals along the length of the chromosome, an alignment density score that is simply the sum of all the lengths of any of the filtered alignments overlapping that interrogation point. A Gaussian kernel density (bandwidth 10 Kbp) was calculated over these interrogation points, weighted by their scores. To allow comparability between windows, the interrogation point densities were normalised by the sum of scores in the window. Runs of interrogation points at which the density surpassed a minimum density threshold were flagged as LDPRs. A few minor adjustments to these regions (merging of overlapping regions, and trimming the end coordinates to ensure the stretches always begin and end in repeated sequence) yields the final tabulated list of LDPR coordinates (Supplementary Tables 1). The pipeline steps following lastz alignment were implemented in R [45] (v4.2.0) making significant use of the package data. Table [46] (v1.14.2; see Code Availability). The pipeline was run on the IPK computing cluster with 16 CPUs, requiring a maximum of approximately 1 TB of memory and running for around 4 CPU-months, with most computing time consumed by the alignment step for windows containing many extreme long runs of very short tandem repeats and near-repeats.

#### Function-agnostic assignment of genes to gene clusters

Primary transcript protein sequences from the MorexV3 annotation [28] were clustered using a global-alignment-based variant of k-means clustering, implemented as the Uclust [47] (v11) algorithm (Supplementary Tables 2). A clustering cutoff of 0.5 proved adequate to ensure that A) the gene cluster sizes were adequately large for statistical power, and B) the collections of functional descriptions given with the MorexV3 annotation found within each gene cluster tended to be overwhelmingly dominated by a single description (i.e., that within a gene cluster, if a combination of the descriptors ‘MADS-box protein’ and ‘MADS-box-family gene’ is found this is acceptable. If broadly differing descriptors are found, this is undesirable).

#### Testing for gene—LDPR associations

We categorised each member of each gene cluster as falling within- or not-within an LDPR based on the annotated coordinates of the coding sequence of the longest transcript of each gene (Supplementary Tables 3). Any overlap was considered sufficient to count the gene as within an LDPR, but we imposed the additional constraint that to be eligible for testing, a gene cluster must contain multiple members within each LDPR. For each gene cluster, we calculated a *p*-value on the null hypothesis that membership in the gene cluster was independent of occurrence within LDPRs (essentially, an urn model) by applying Fisher’s exact test (two sided) (Supplementary Tables 4). Since only non-singleton gene clusters were eligible to show significant association with LDPRs, we chose an appropriate p-value cutoff to indicate significance accounting for multiple testing using the Bonferroni correction, i.e. 0.05 divided by the number of gene clusters possessing more than one member = 0.05/6,419 ≈ 7.79 × 10^–6^.

#### Testing for evidence of selective action using gene function information

We required a pool of ‘test’ genes likely to be enriched in functions conferring a selective benefit in arms races. To ensure maximal objectivity, we assigned pool members strictly based on compilations and assignments from the literature and public databases. First, we selected all genes whose GO ontology terms fell under the parent descriptor GO:0044419 “biological process involved in interspecies interaction between organisms”. We added to these genes any with homology-based gene descriptors matching the list of domain descriptors shown to play a role in pathogen resistance in barley and at least one other cereal species, compiled by Krattinger & Keller [48] (See Supplementary Notes 2 for details on the regular expression used for matching human-readable descriptors). Any gene clusters having greater than 50% of its members in this pool (*n* = 458 of 17,188 gene clusters) was assigned to the probable-arms-race pool. Only 17 of these 458 gene clusters had any members not in the pool. We then tested for significant overlap by fitting a logistical regression model (arms race pool membership ~ strength of evidence of association with LDPRs [-log(*p*-value)]), assessing the predictive power of LDPR association on arms race pool membership with a likelihood ratio test (Supplementary Tables 5).

#### Method validation using housekeeping genes

The methods described in the previous two sections were applied identically to test whether housekeeping genes associate with duplication-prone DNA, with the following changes: Instead of a list of gene clusters putatively enriched in arms-race genes, we created an analogous list of clusters putatively enriched in housekeeping genes. We identified such genes beginning with the CDS sequences of housekeeping genes nominated as expression standards by Gines et al. (2018) [35], which we accessed at the archived uniprot ftp site https://ftp.ncbi.nlm.nih.gov/repository/UniGene/Hordeum_vulgare/. Homologous genes from the MorexV3 annotation were found using a homology search with BLASTn (v2.14.0; default parameters), and any genes for which any splice variant achieved an alignment covering over 80% of the combined original sequences were labelled as putative housekeepers. As before, clusters with > 50% putative housekeeper members were labelled housekeeper clusters. The rest of the pipeline follows identically to that described above.

#### Phylogenetic trees for gene clusters

Protein sequences for gene clusters of interest were multiply aligned using MUSCLE (v3.8.31, default parameters) and maximum likelihood trees were constructed using PHYLIP (v 3.696, default parameters).

#### Possible functional effects of particular gene clusters under study

While not the main focus of this study, we conducted short investigations to inform speculation on the function of the specific gene clusters that we used as representative examples (Supplementary Notes 1, Supplementary Data, and Supplementary Tables 6). We examined protein structural predictions (ColabFold [49]), the EoRNA Barley gene expression database [50], and pan-tissue PacBio IsoSeq transcript sequences [51] aligned to the MorexV3 genome [28] using BLASTn (v2.10.0; default parameters).

#### Investigation of specific LDPRs

Members of the twenty gene clusters with the strongest evidence of LDPR association (by p-value) were grouped into ‘regions’ wherever they fell within 1 Mbp of each other. For each gene cluster, the regions were pairwise aligned with as described above under “*Gene-agnostic identification of long-duplication-prone-regions*”. The alignments were parsed and plotted using custom scripts making extensive use of R base::plot and data.table functions (versions as above). Three particularly informative cases that demonstrate general trends seen across these twenty gene clusters (corresponding to cl_1888, cl_8856, and cl_16606) were selected as the basis for discussion in the manuscript, the remainder are shown in Supplementary Materials 1.

## Supplementary Information

Supplementary Material 1.

Supplementary Material 2.

Supplementary Material 3.

Supplementary Material 4.

## Data Availability

All data used are publicly available and links to the associated papers are given in the relevant parts of the text and bibliography. Data produced by the analysis are given in the Supplementary Tables. The code used to perform the analyses described is available at github.com/mtrw/RGS.
