# Supplementary material for: Do duplication-inducing elements ‘cooperate’ with genes in evolutionary arms races? A case study on cereal crop pathogenesis
Source: BMC Plant Biol. 2025 Oct 30;25:1478. doi: 10.1186/s12870-025-07328-6 (PMC12573847; doi:10.1186/s12870-025-07328-6)
Supplement: Supplementary file 1 — Supplementary Material 1. [file 12870_2025_7328_MOESM1_ESM.pdf]

SUPPLEMENTARY NOTES

Supplementary Note S1. *Supplementary note describing investigations into the possible functionality of genes discussed in the main study.*

Testing for association between arms-race-implicated gene clusters and l-DPRs (see main text methods and results), the lowest p-value is attained by cl_16606, which has mixed descriptions as a result of this short (99 residues for all members) predicted protein's general lack of homology to any functionally-characterised gene (the most common descriptor being 'T-box transcription factor', a gene exclusive to animals), and is likely an unexpressed or lowly-expressed pseudogene. Inference of any cl_16606 members' protein structure using the ColabFold (accessed 23rd June, 2022) with default parameter settings, recovers no discernible canonical secondary structures. An expression profile database that features the reference cultivar 'morex'[1–3] contains several expressed genes containing 100% matching alignments to the whole length of cl_16606 CDSs (SM tables 6), but all these genes contain a greater number of exons than the single exons of each annotated cl_16606 member. This is confirmed by sequence data directly: searching for cl_16606 members with 100% matching alignments (blastn 2.9.0; default parameters) to PacBio HiFi Iso-Seq-derived transcript sequences[1] (Supplementary Table 6) results in transcripts of minimum length 874 bp (read m54203U_210322_143606/163908242/ccs), all of which have several in-frame stop codon sequences downstream of that corresponding to the cl_16606 predicted protein.

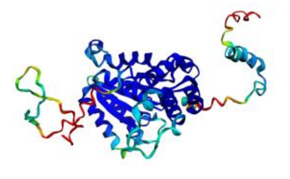

Predicted tertiary structure of a representative member of cl_1888.

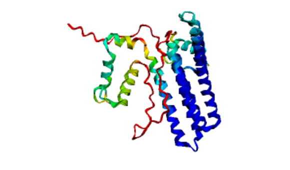

Predicted tertiary structure of a representative member of cl_8856.

In contrast to the likely-inactive cl_16606 genes, there is ample evidence that clusters cl_1888 (fig 3) and cl_8856 (fig 5) do or did possess biological function. Members of both show confidently-predicted secondary protein structure, and ample Iso-Seq-derived transcript sequences containing 100% matches to their full lengths with under 1 Kbp of 5' UTR and polyadenylation signals downstream (cl_1888: n=1; cl_8856: n=8; Supplementary Table 6).

In the MorexV3 annotation, Cluster cl_1888 members are described as most similar to the poorly-researched ATPase 'cell-division cycle 48–like', and possess sequence homology to Arabidopsis genes implicated in protein degradation, cell-cycle control, gene activation, and the turnover of NLR immune receptors`[4–7]. Transcripts in the EORNA barley expression database with 100% matches to members and not exceeding the length of the CDS by 900bp are expressed in both coleoptile and pollen tissues (Supplementary Table 6).

Cl_8856 are annotated in MorexV3 as most similar to 'Jasmonate-induced protein', a class of proteins implicated in activating herbivore or pathogen defence responses[8]. Transcripts in the EORNA barley expression database with 100% matches to members and not exceeding the length of the CDS by 900bp are show evidence for expression in coleoptile, apical meristem, and various spike and leaf tissues (Supplementary Table 6).

Supplementary Note S2. *Genes selected for the arms race pool based on human-readable descriptors were matched by the following POSIX-compliant regular expression.*

NBS.*LRR(-| |$)|NLR(-| |$)|TLR(-| |$)|[Tt]oll[-][Ll]ike|[Ll]eucine.(Rr)ich.(Rr)epeat|RLK(-| |$)|rlk(-| |$)|([Rr]eceptor[-]|[Pp]rotein[-]).*[Kk]inase|[Rr]eceptor[-][Llike][-][Pp]rotein|[Ll]ectin[- $]|[Jj]acalin[-]|[Tt]hioredoxin(-| |$)|WRKY[-]|BED[-]|FNIP[-]|[Kk]elch[-]|[Pp]rotein[-][Pp]hosphatase[-]2C|[Pp]athogen|[Gg]lutaredoxin|DDE[-]|[Ee]xo70|[Dd]isease|[Rr]esistance
