# Supplementary material for: Do duplication-inducing elements ‘cooperate’ with genes in evolutionary arms races? A case study on cereal crop pathogenesis
Source: BMC Plant Biol. 2025 Oct 30;25:1478. doi: 10.1186/s12870-025-07328-6 (PMC12573847; doi:10.1186/s12870-025-07328-6)
Supplement: Supplementary file 2 — Supplementary Material 2. [file 12870_2025_7328_MOESM2_ESM.pdf]

SUPPLEMENTARY FIGURES

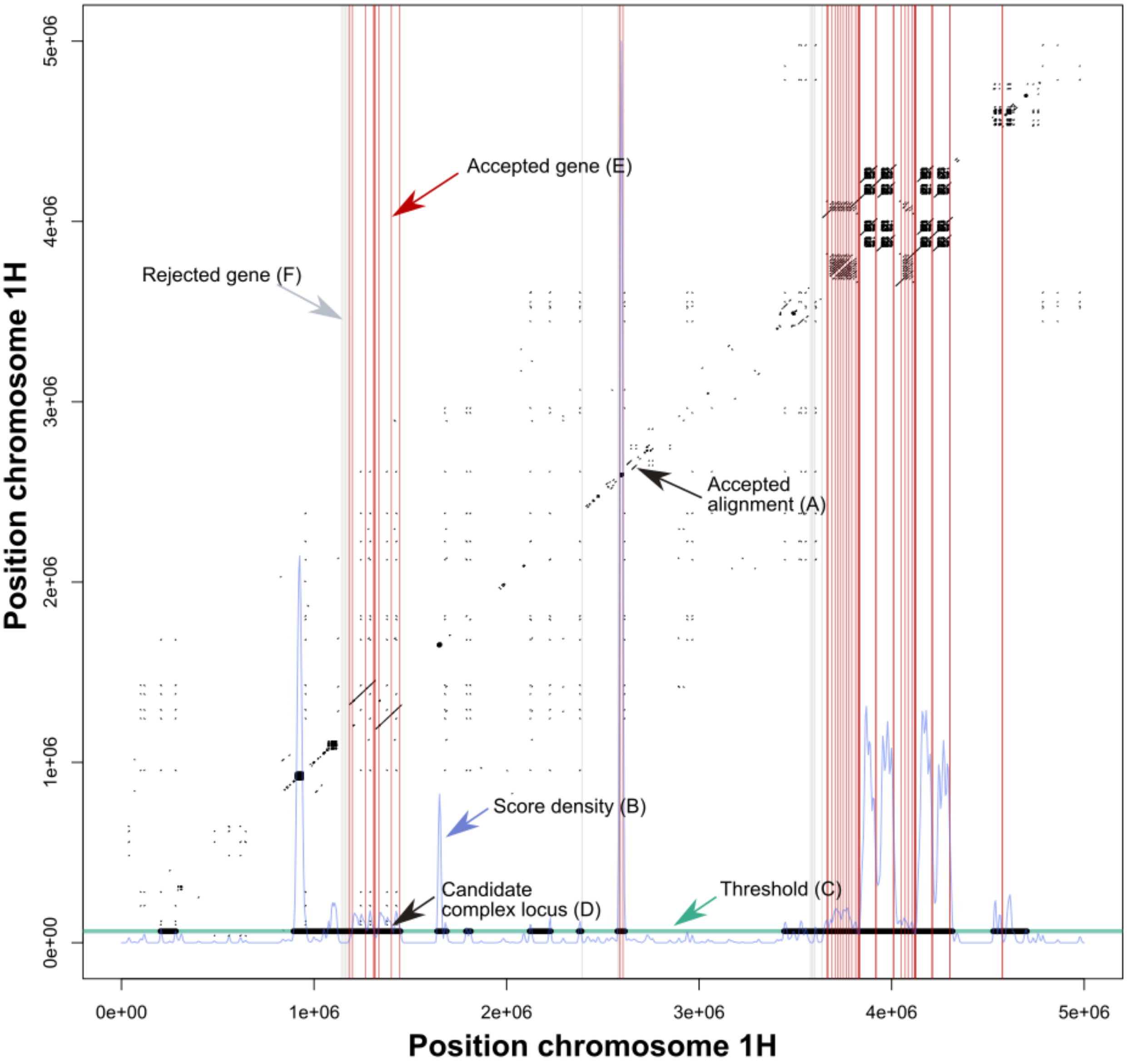

Supplementary Figure S1. **Visualisation of the algorithm for identifying candidate l-DPRs, demonstrated on a 5 Mbp window of chromosome 1H.** Self-aligned parts of the sequence above a particular are shown in black (A; the trivial diagonal alignment is omitted, and alignments are only included that exceed 1.5 Kb in length and for which the aligned regions occur within 1.5 Mbp of one and another—note the latter constraint causes only alignments within). The density of alignments across the region (weighted by their lengths) is shown with a blue line (B). Since the The total mass of this density function is normalised to correspond to the total length of filtered alignments in each window. The density threshold used to flag regions as potential l-DPRs is represented by a green bar (C). The final l-DPRs after merging and trimming candidate regions are shown in thick black (D). The positions of genes are marked with vertical red bars. To be counted as 'within an l-DPR' for the purposes of association testing (E), the gene had to occur within an accepted alignment, and more than one member of the gene's cluster had to occur within the same l-DPR. Genes within l-DPRs but rejected for association testing are shown with grey bars (F).

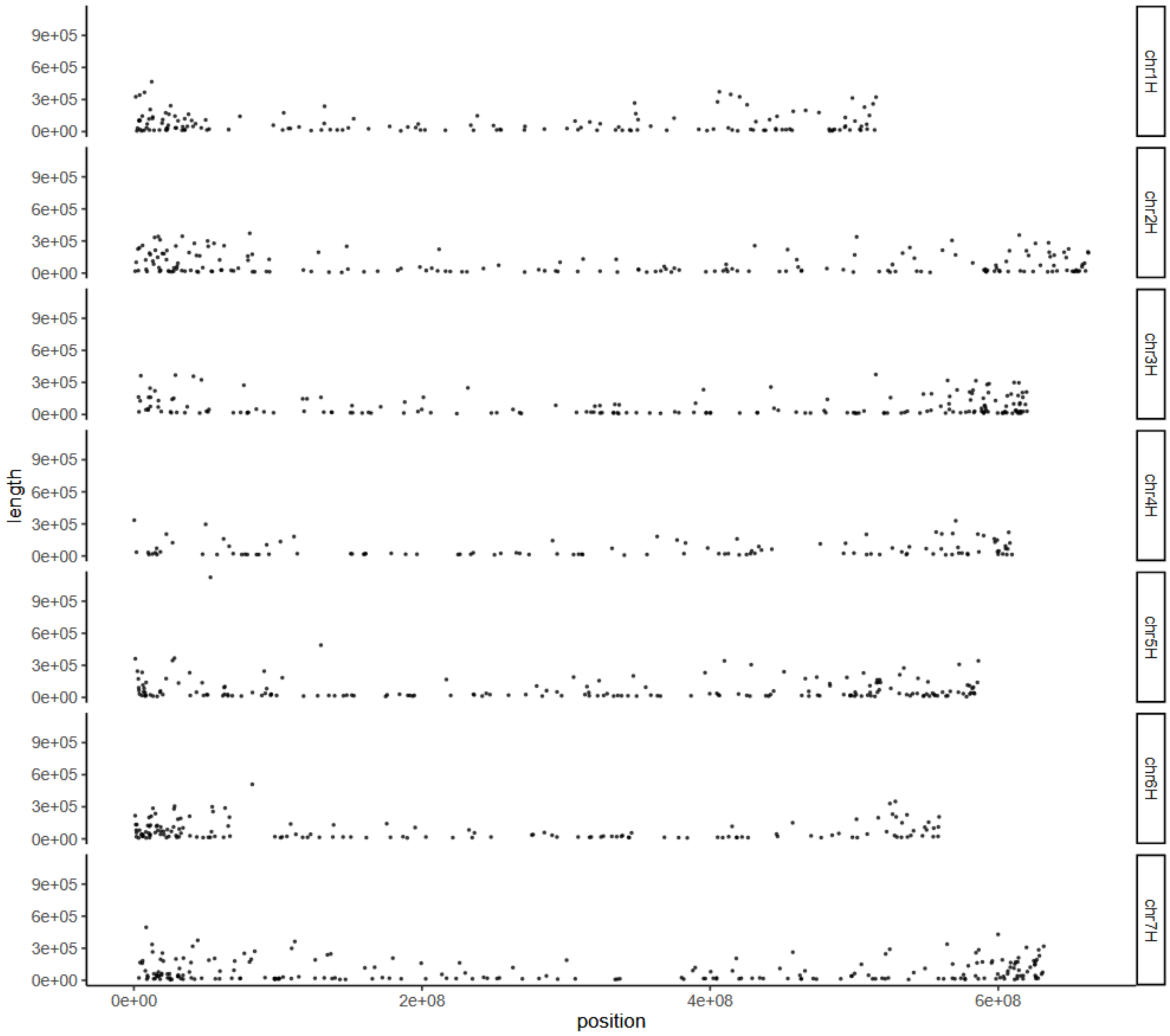

Supplementary Figure S2. **Positions of LDPRs on the MorexV3 barley reference genome, showing LDPRs occur most frequently in subtelomeric zones**. LDPRs located along chromosomes chr1H to chr7H are indicated by points. Length (y-axis) refers to the length of the LDPR region. The subtelomeric zones in which most LDPRs occur also correspond with the regions with the highest density of genes, and the highest frequency of recombination events.

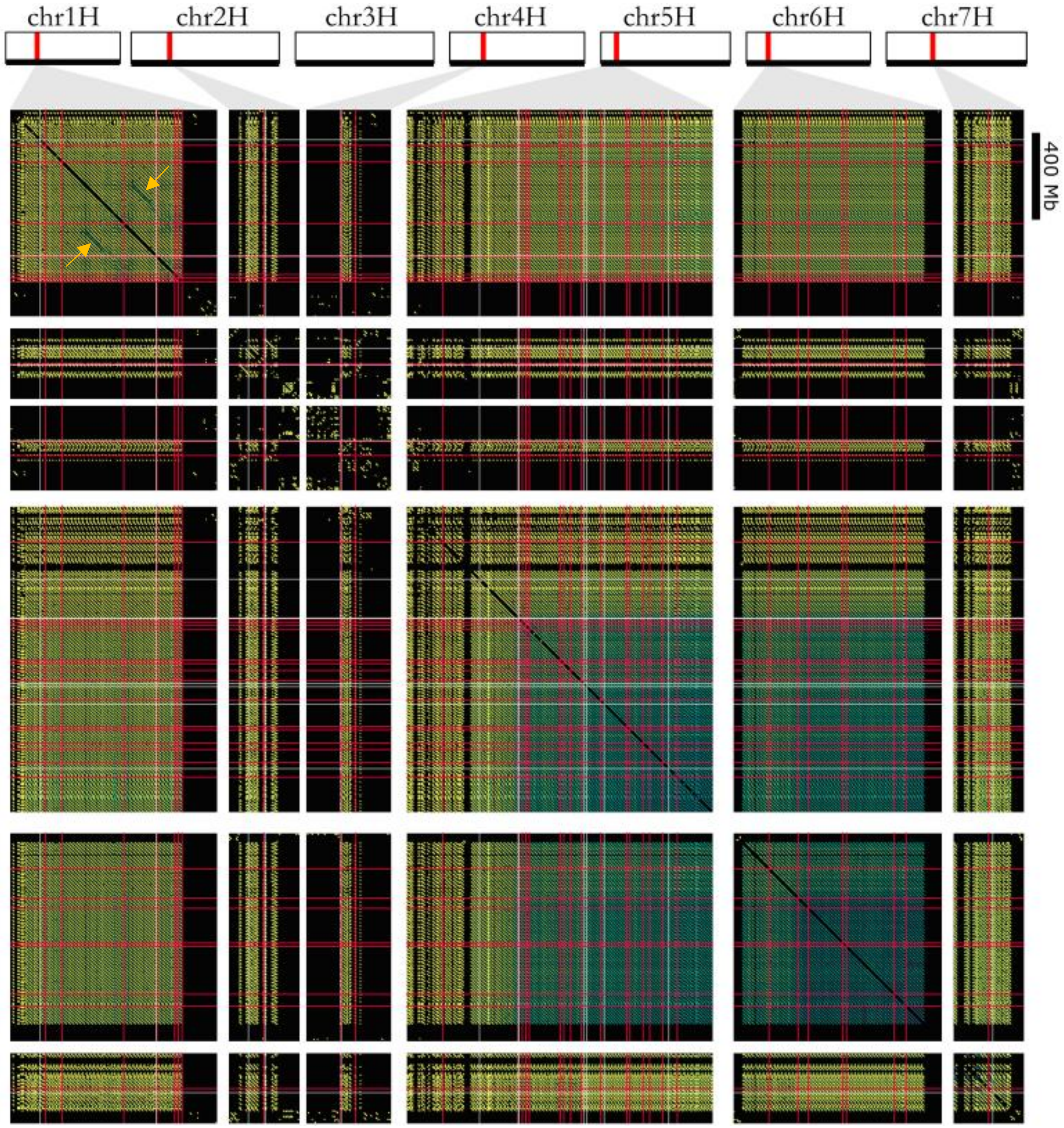

Supplementary Figure S3. **Alignment plot showing related LDPRs**, containing gene cluster B (cl_16606), which occurs on six chromosomes. Figure features follow the analogous panel in main text figure 4, with the yellow—to—blue continuum denoting progressively more similar alignments. Arrows point to instances where greater homology is indicated between runs of repeats somewhat remote from each other, indicating where a group of tandem repeats have likely been recently copied together—discussed in the main text. In contrast with most other observed LDPR-associated gene clusters, cl_16606 has retained much of the repeating unit that constitutes its main duplication-inducer.
