# Supplementary figures and images for: Do duplication-inducing elements ‘cooperate’ with genes in evolutionary arms races? A case study on cereal crop pathogenesis
Source: BMC Plant Biol. 2025 Oct 30;25:1478. doi: 10.1186/s12870-025-07328-6 (PMC12573847; doi:10.1186/s12870-025-07328-6)

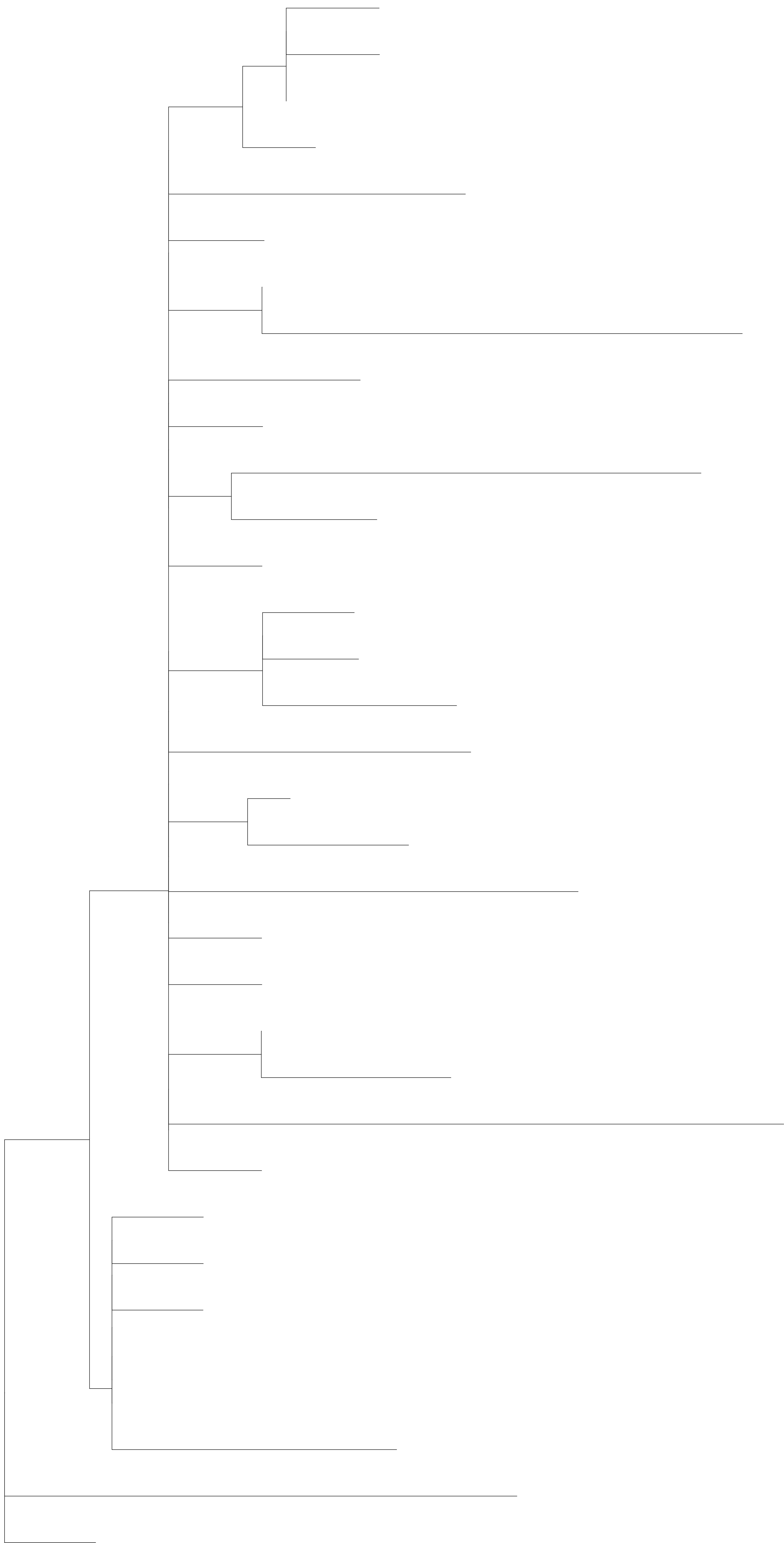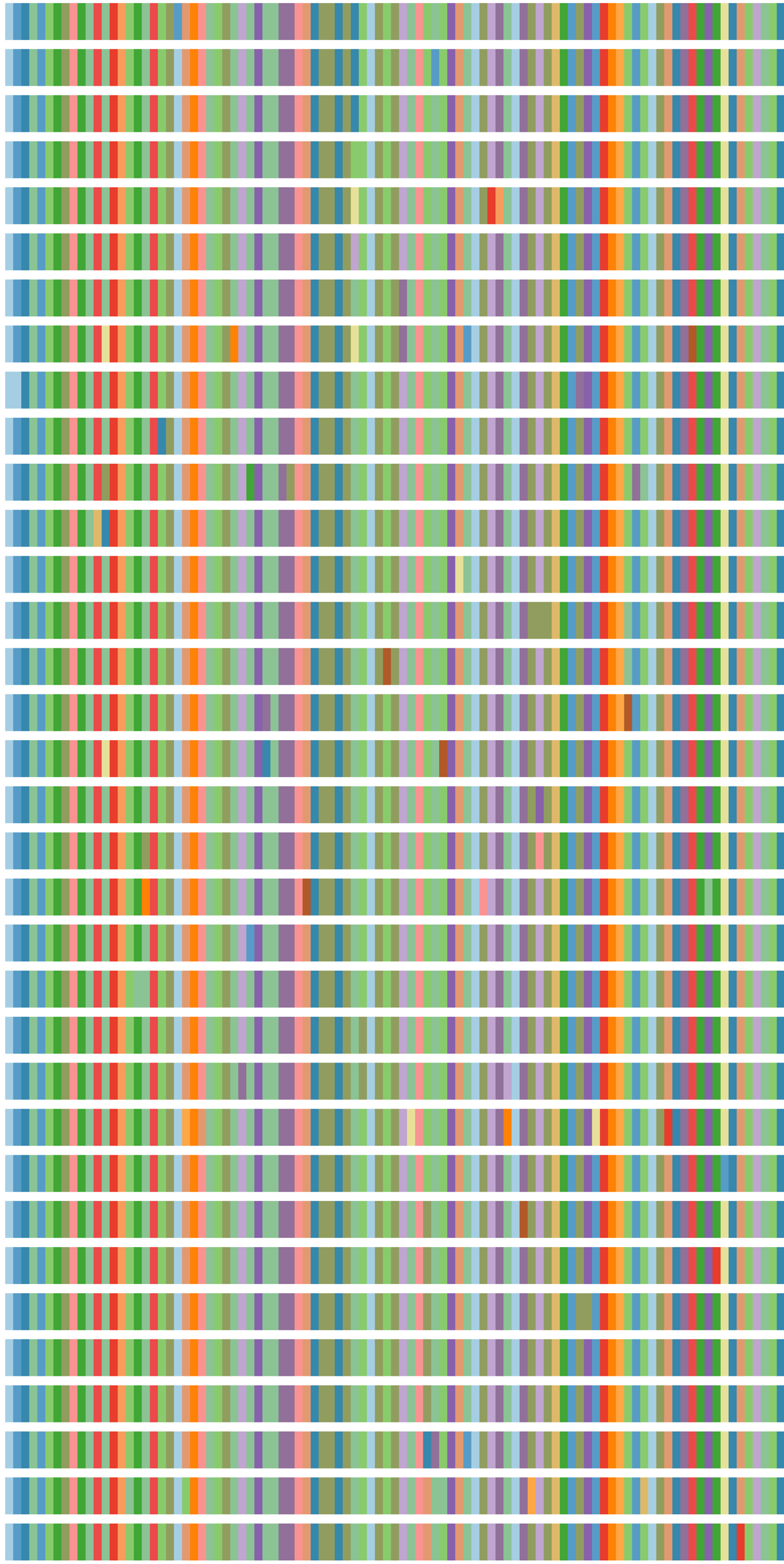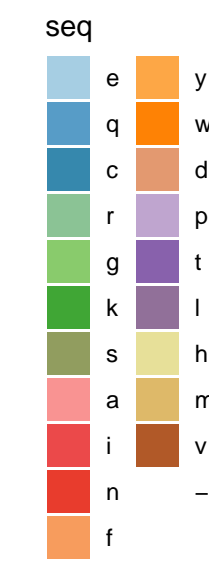



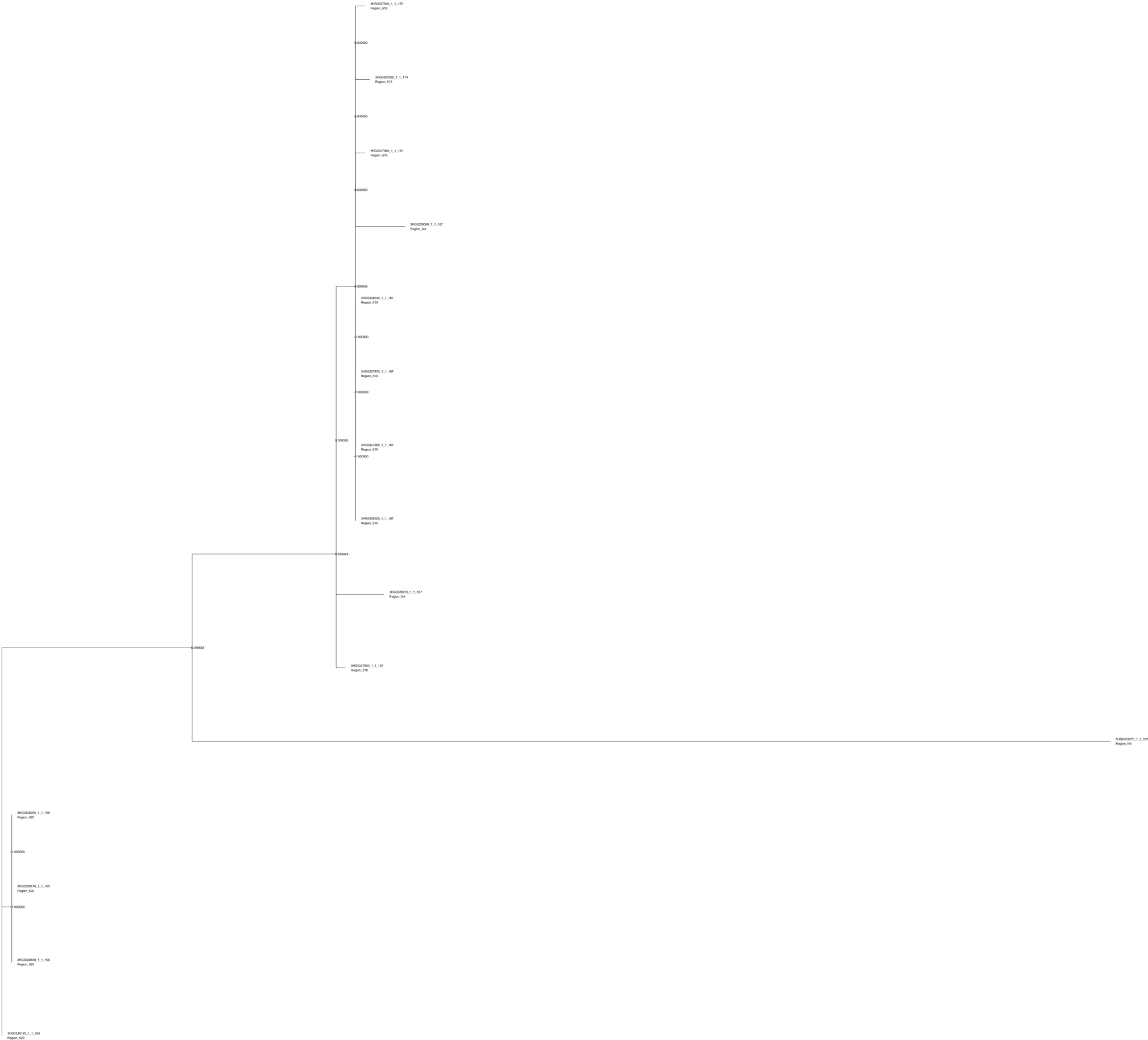

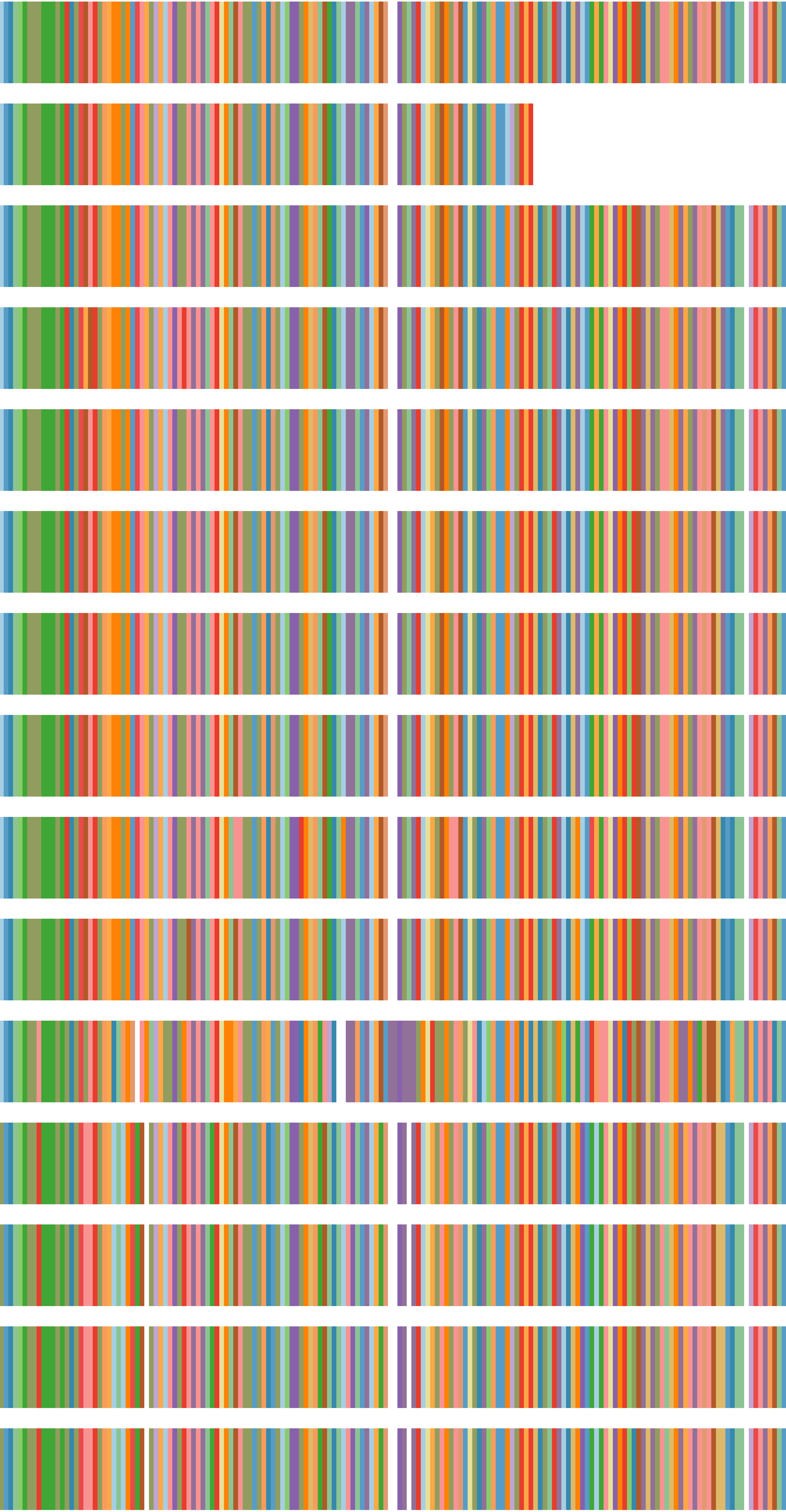



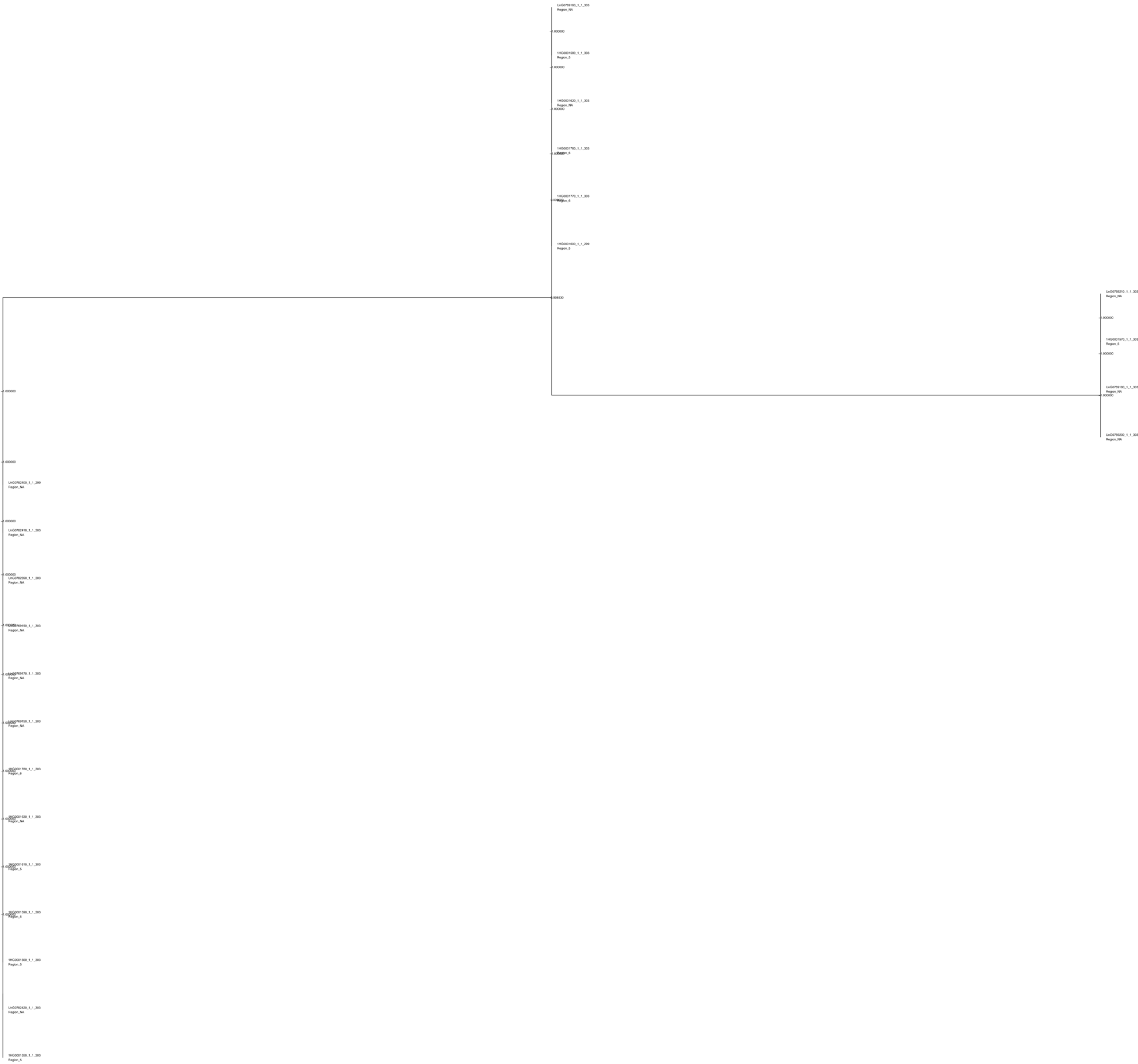

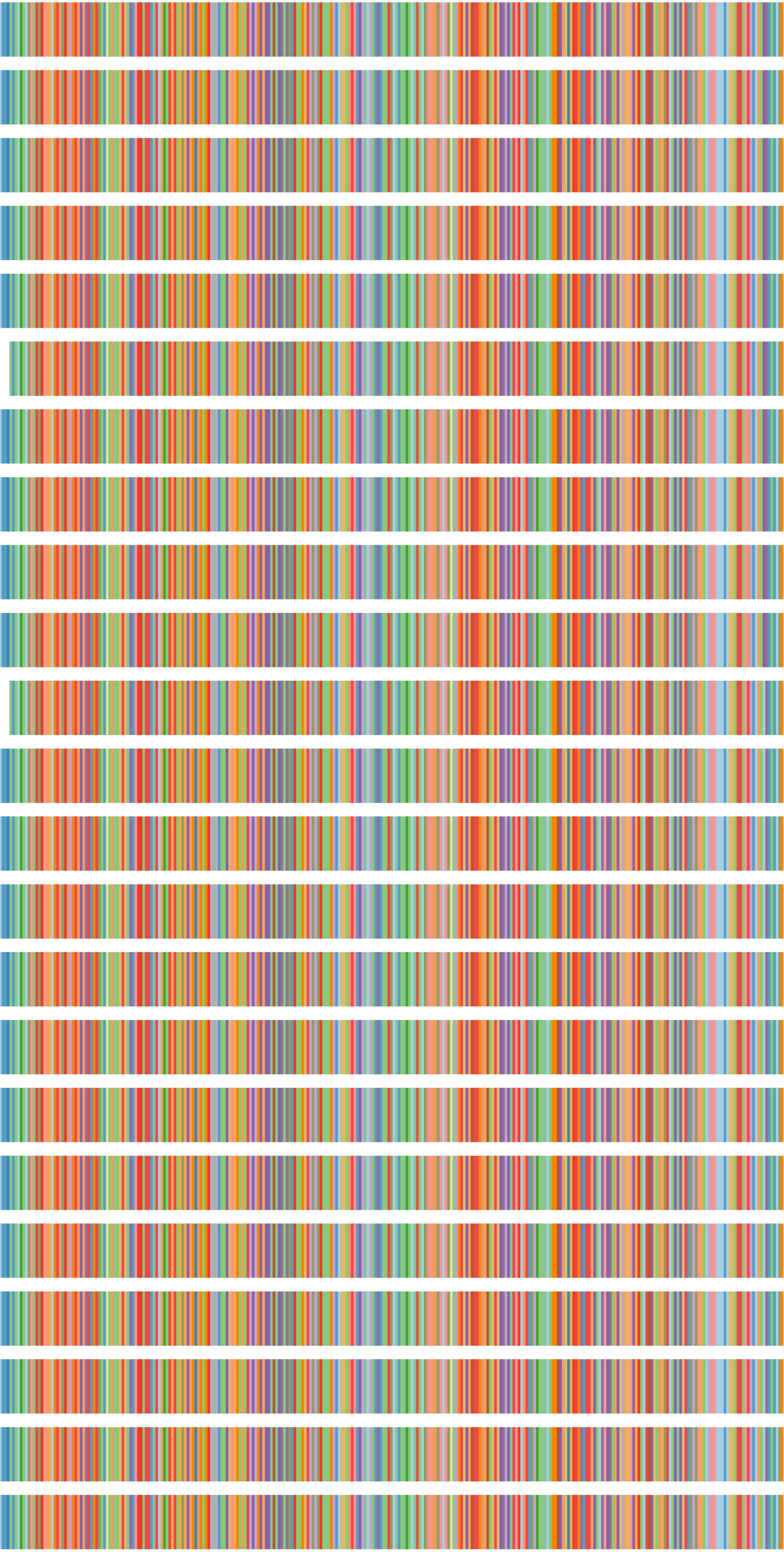



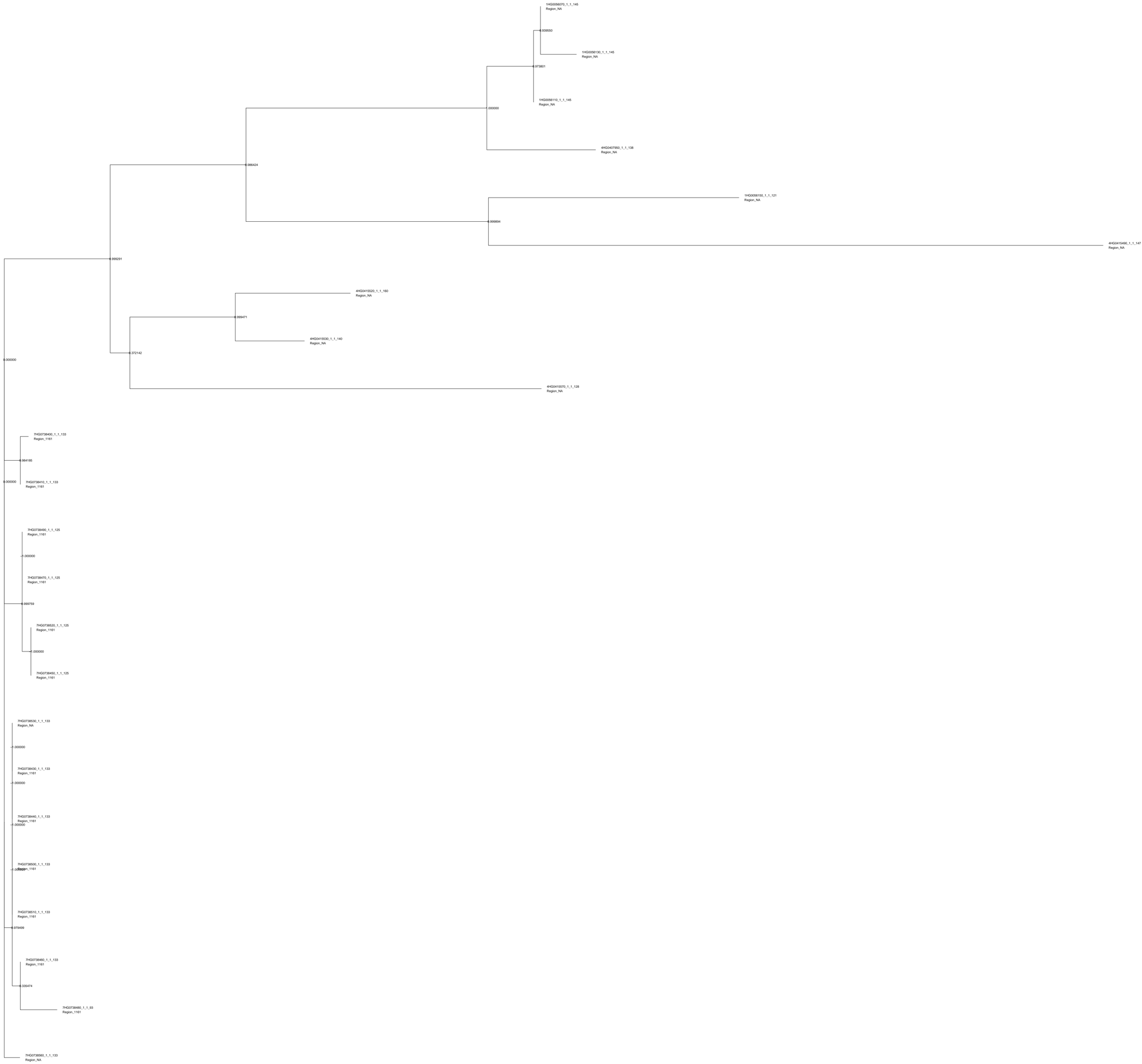

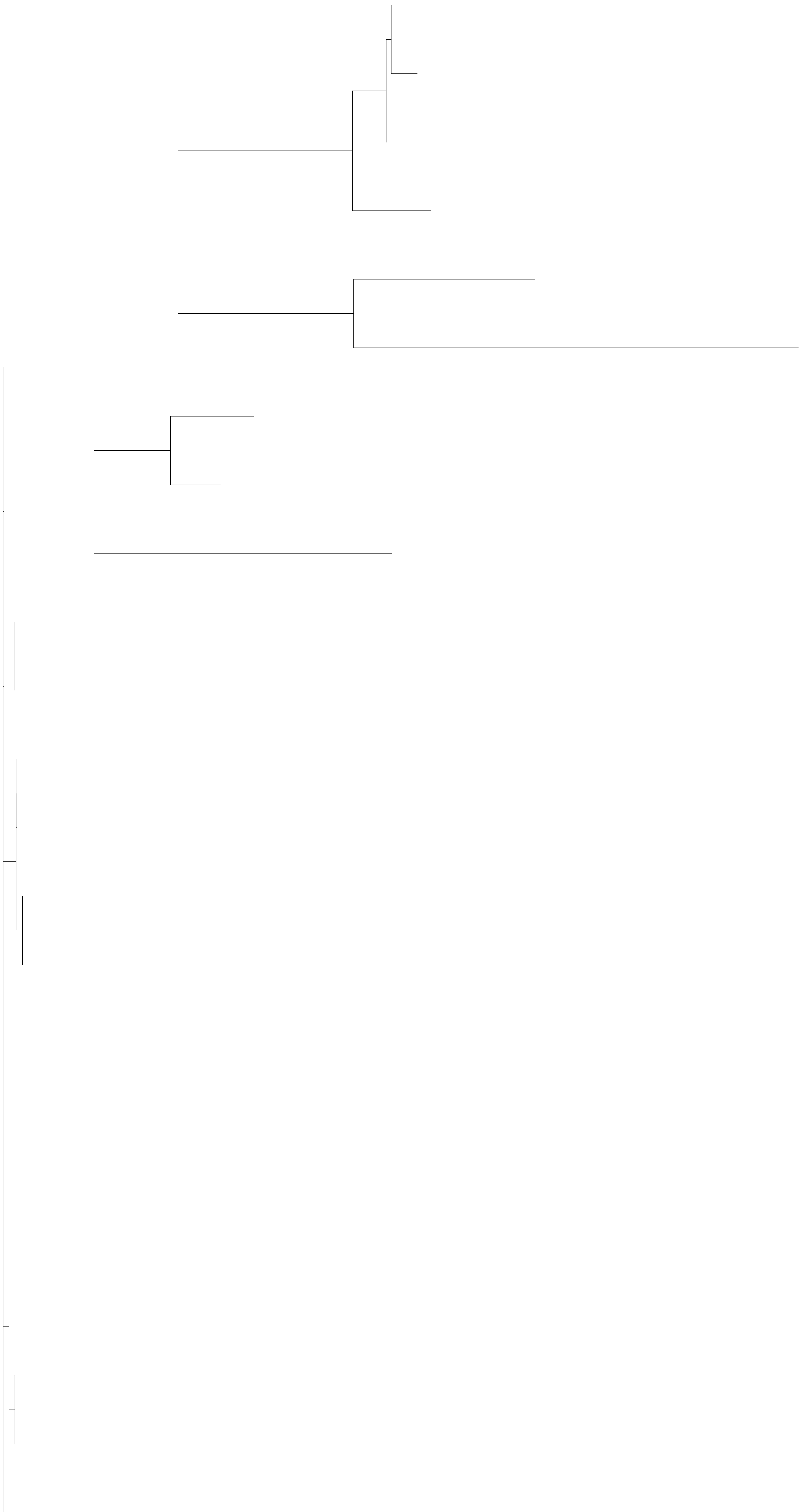



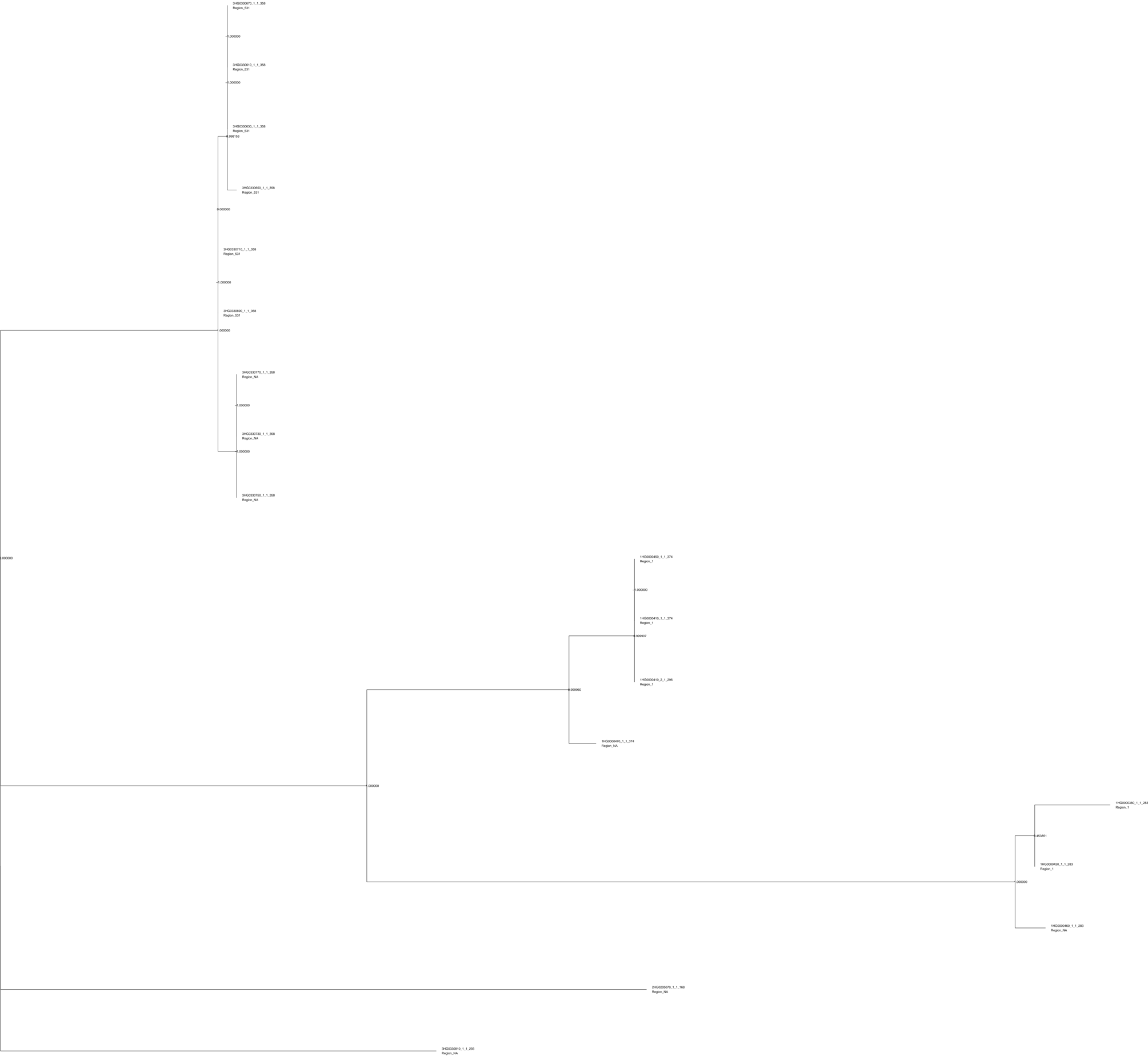

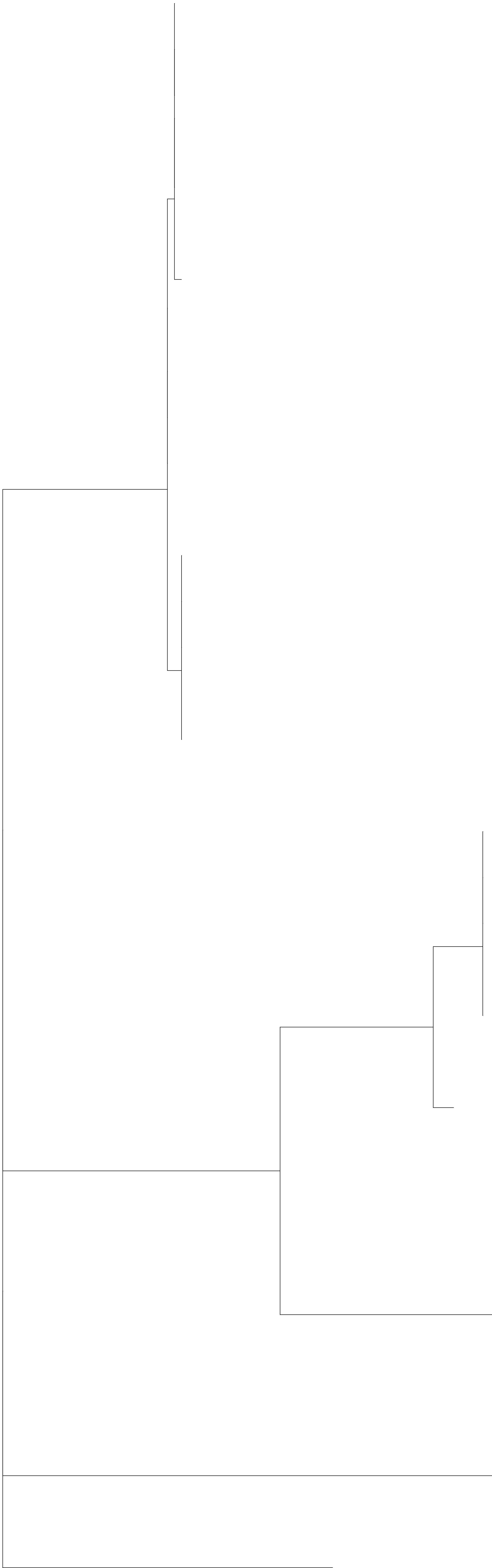



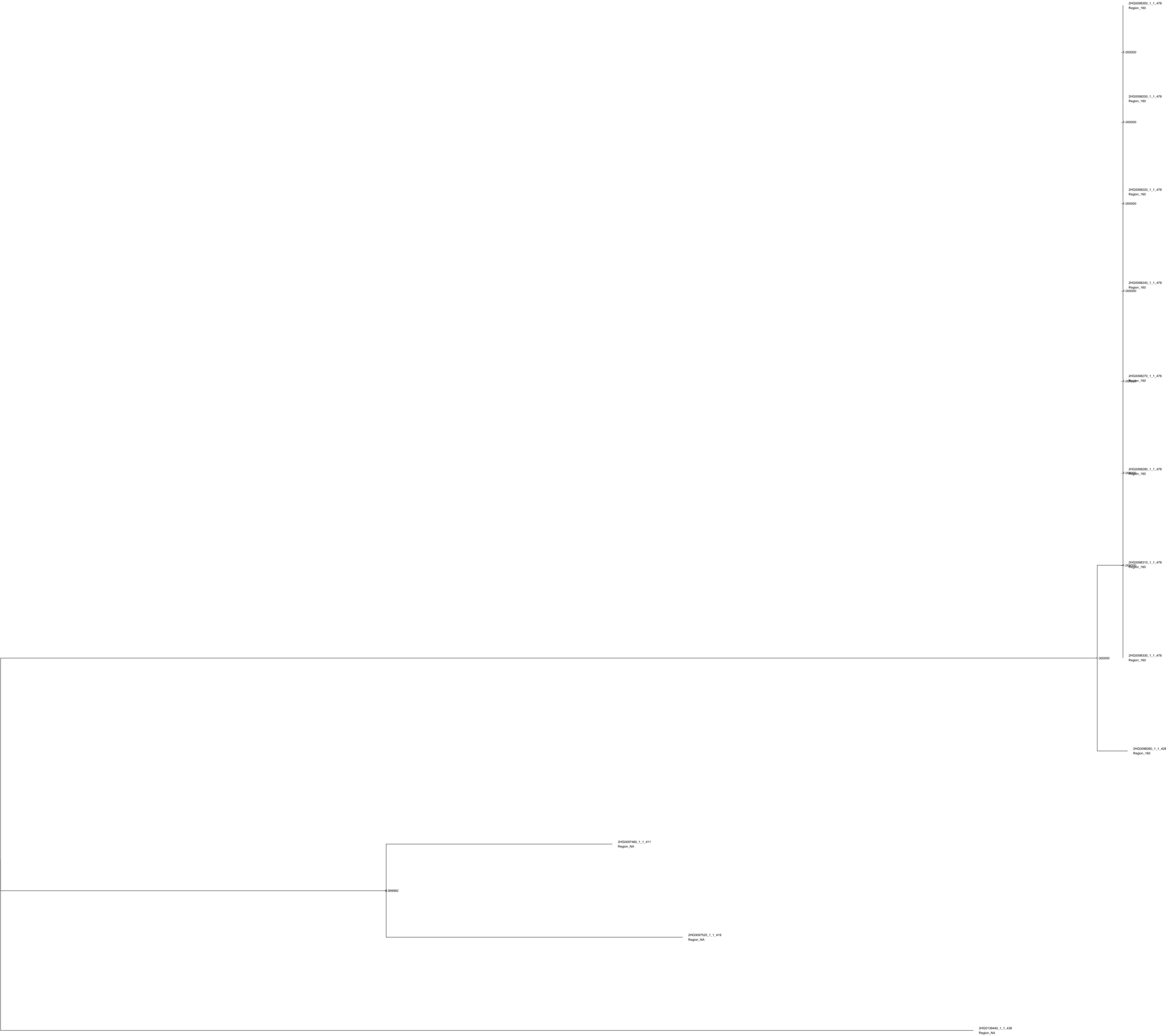

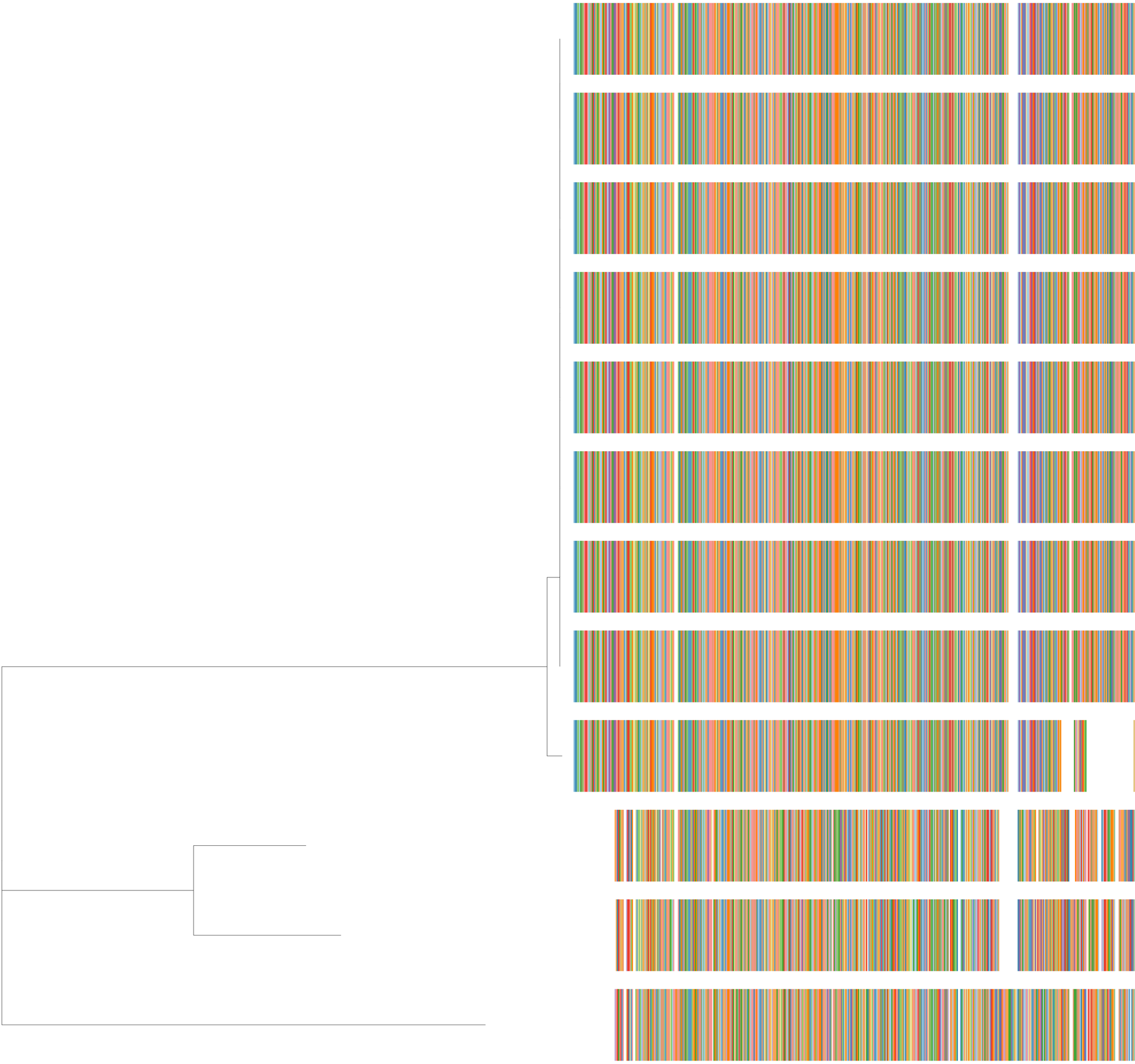



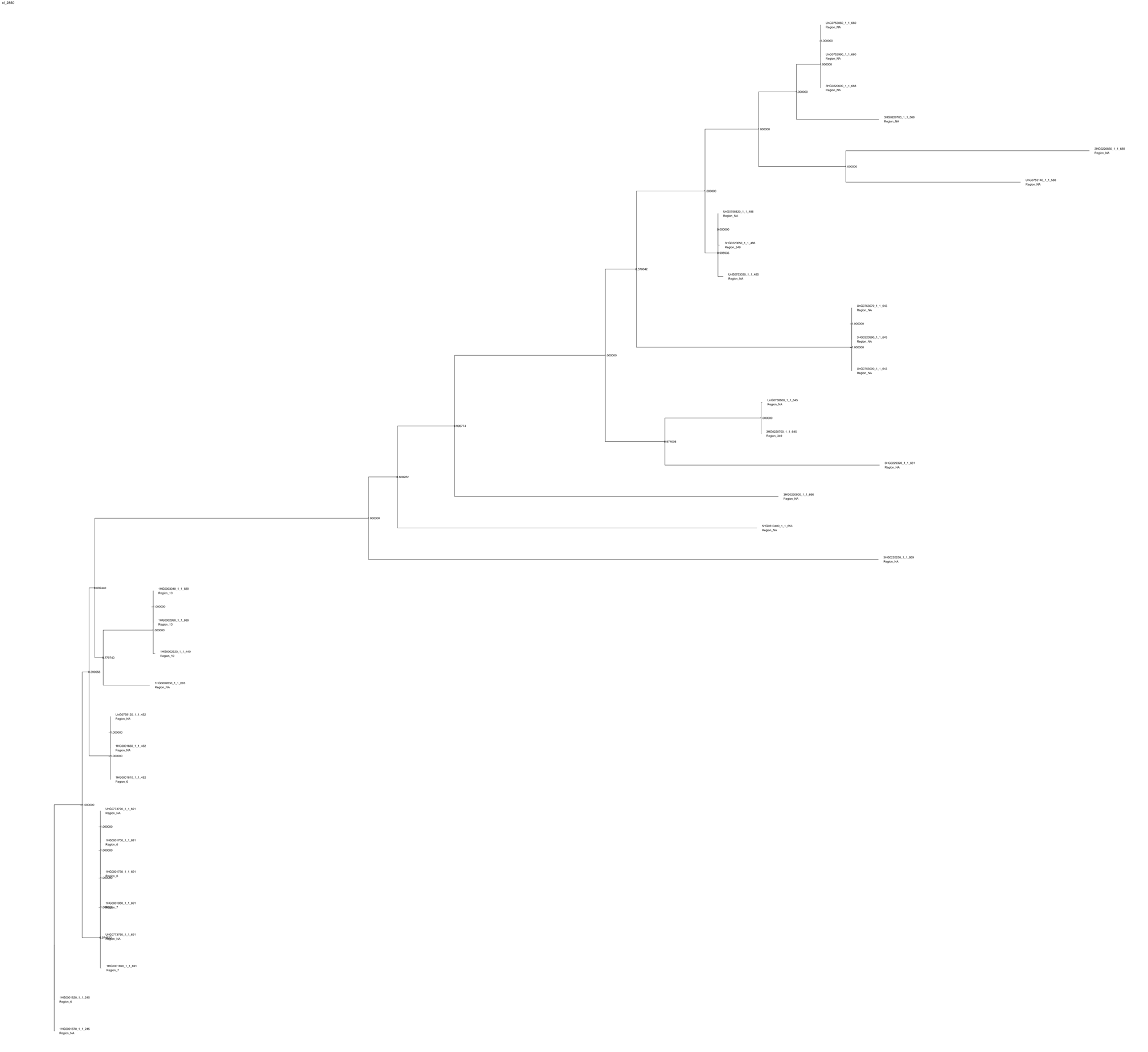

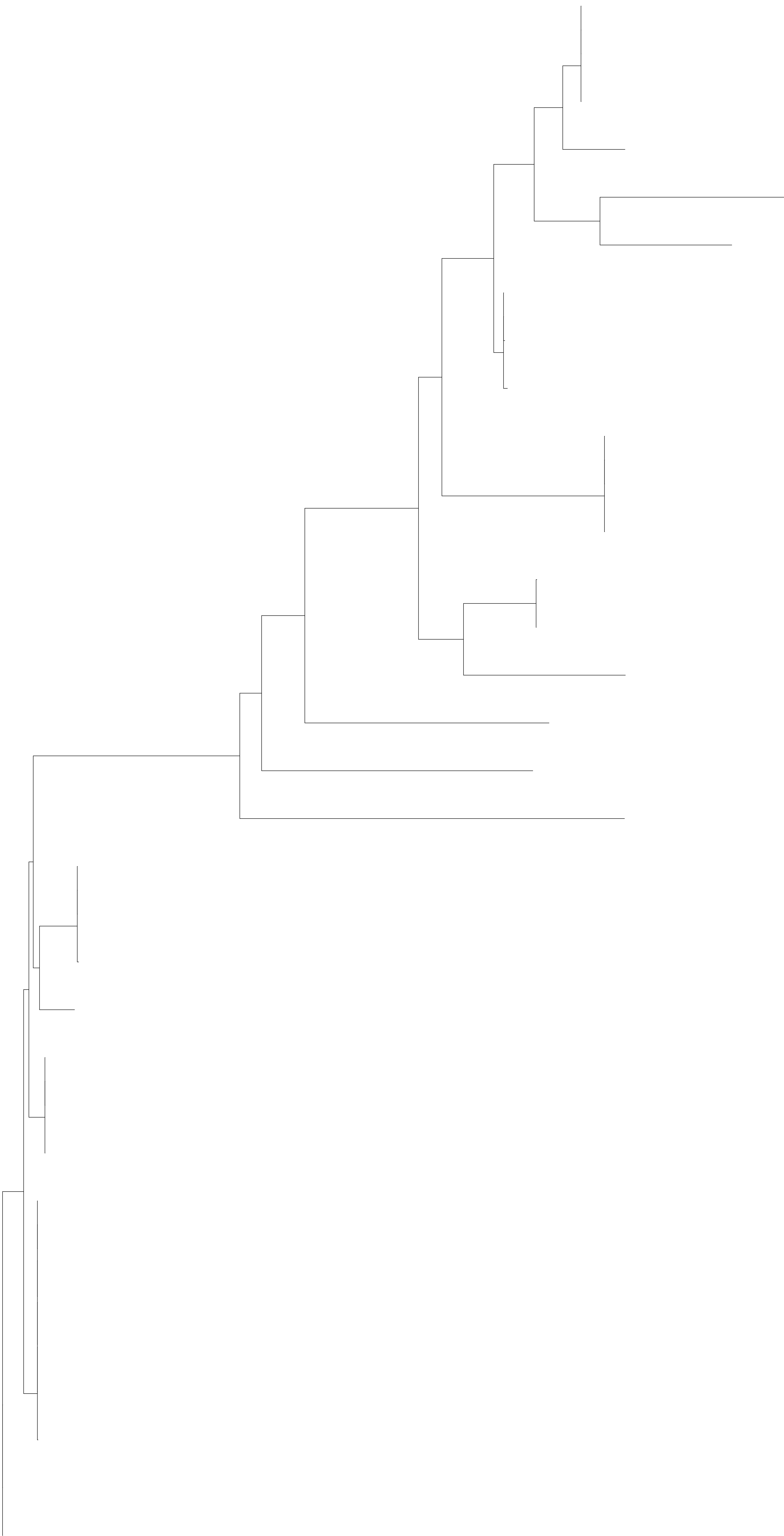





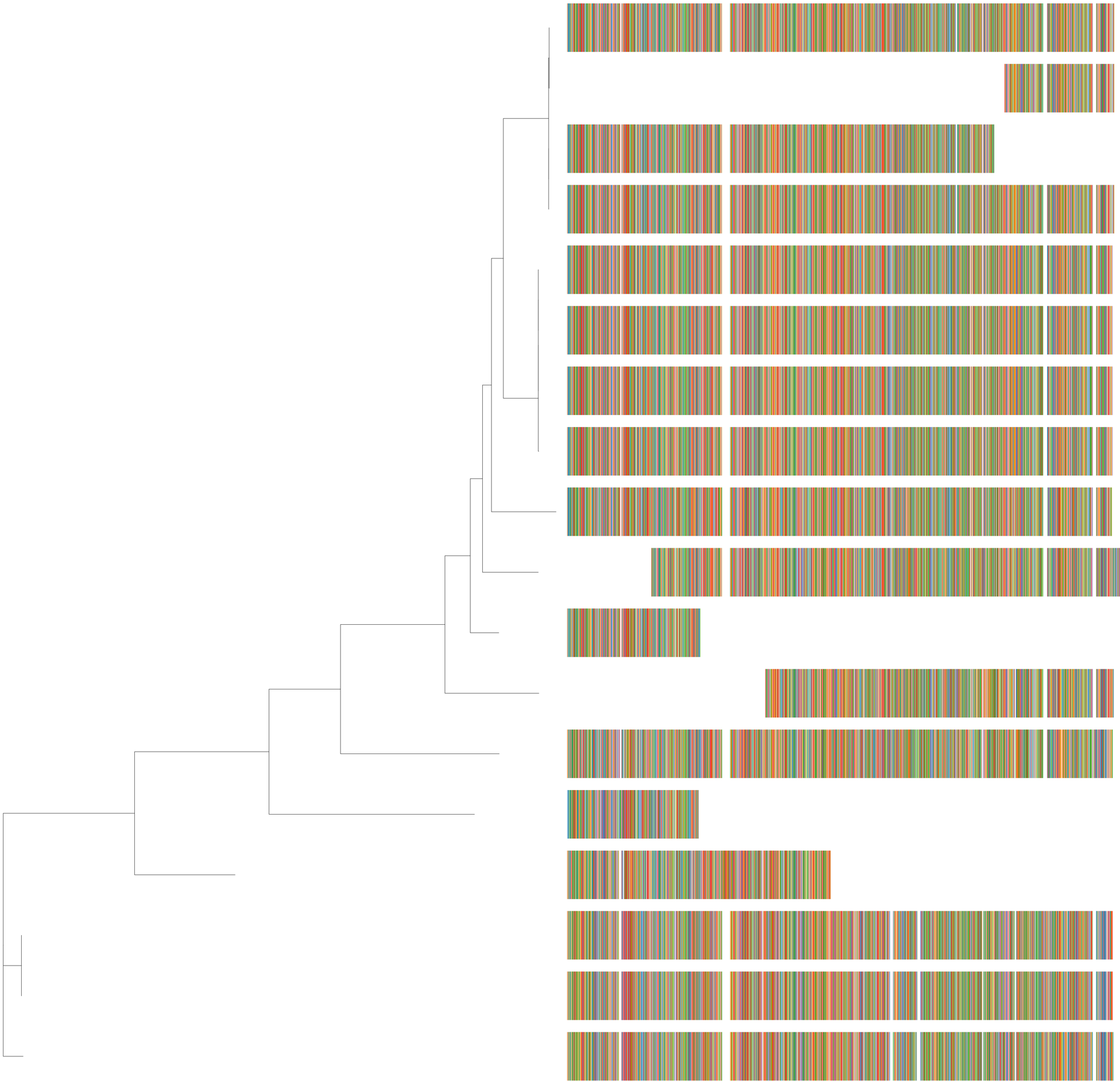





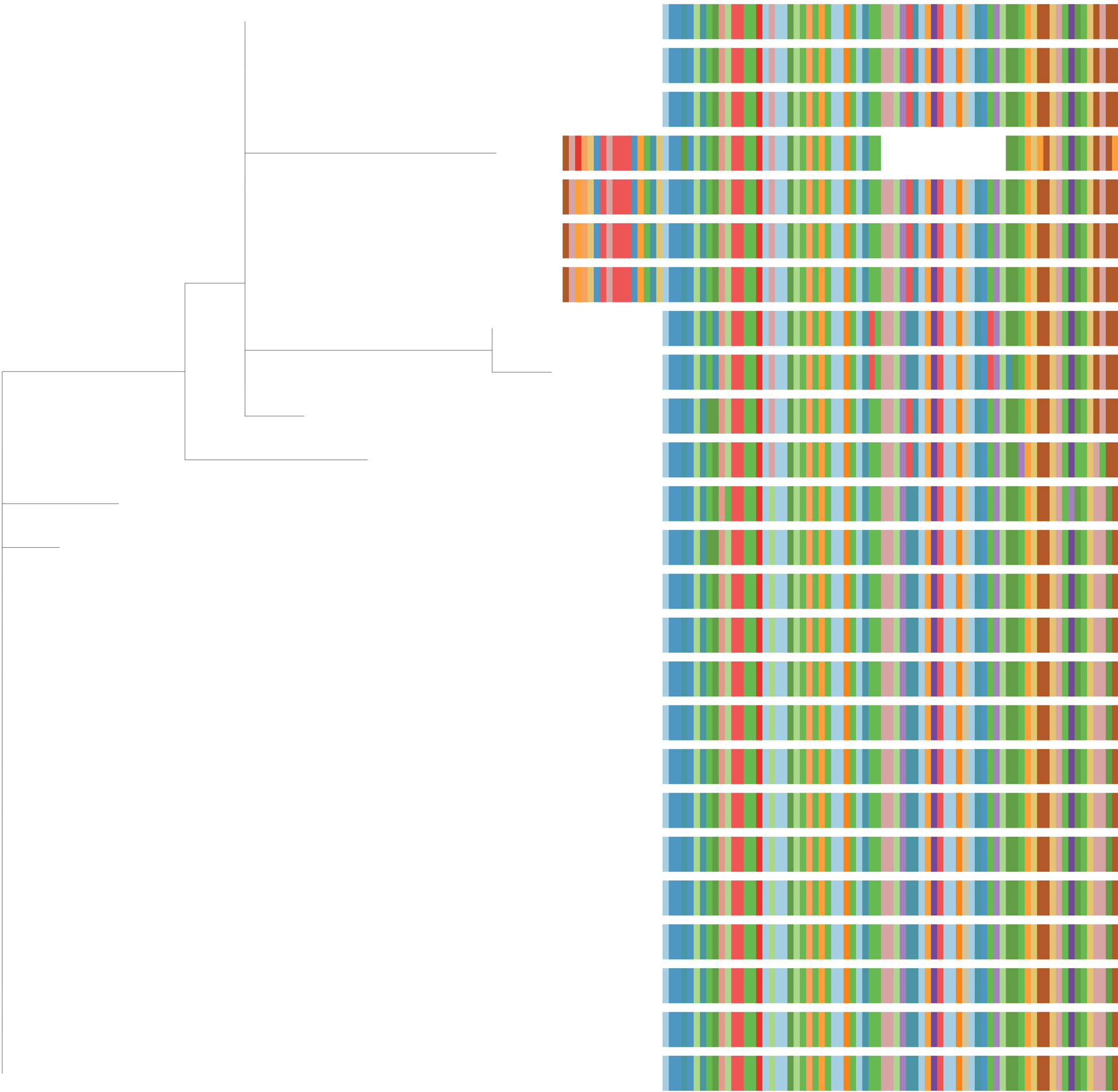





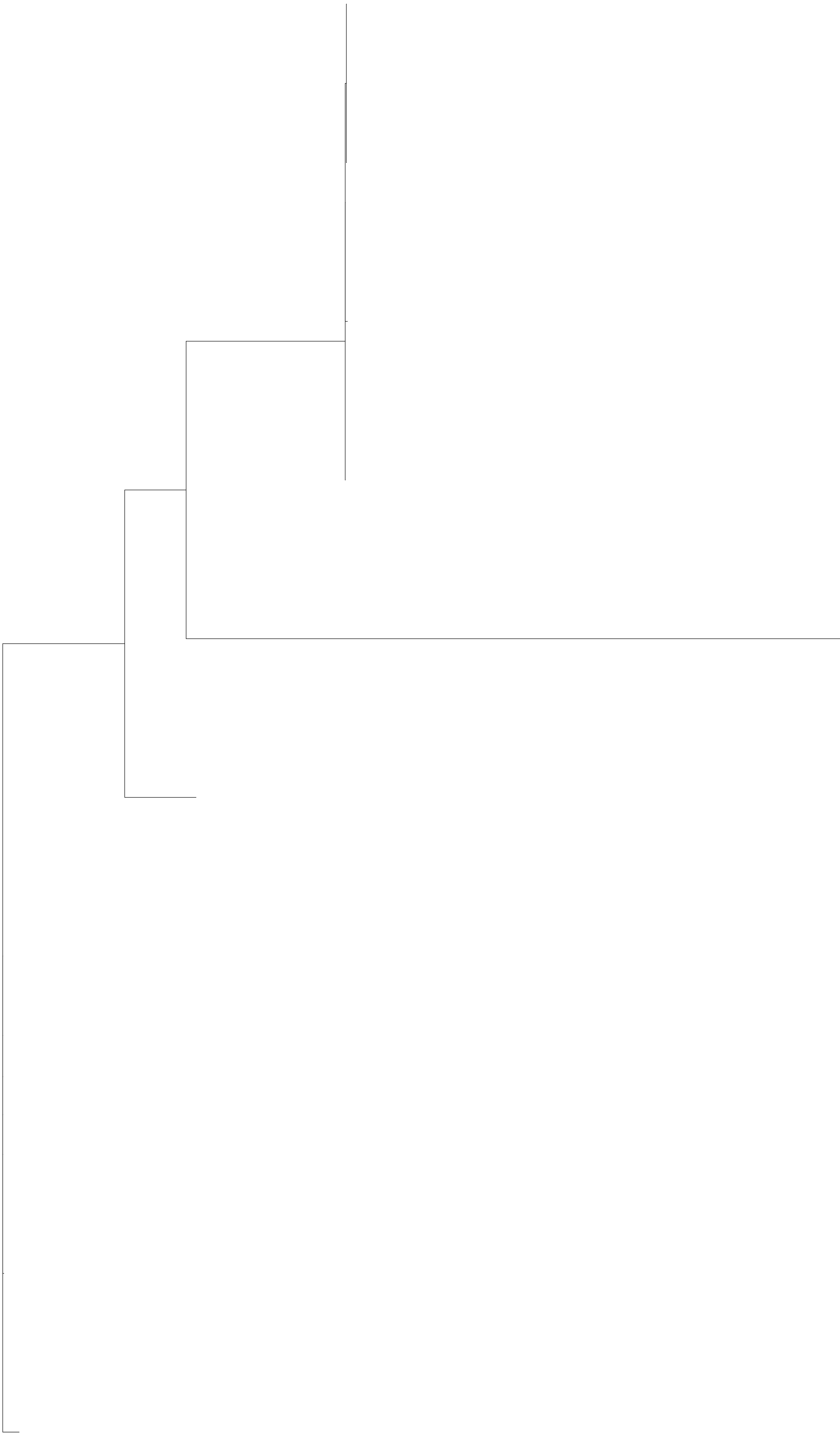





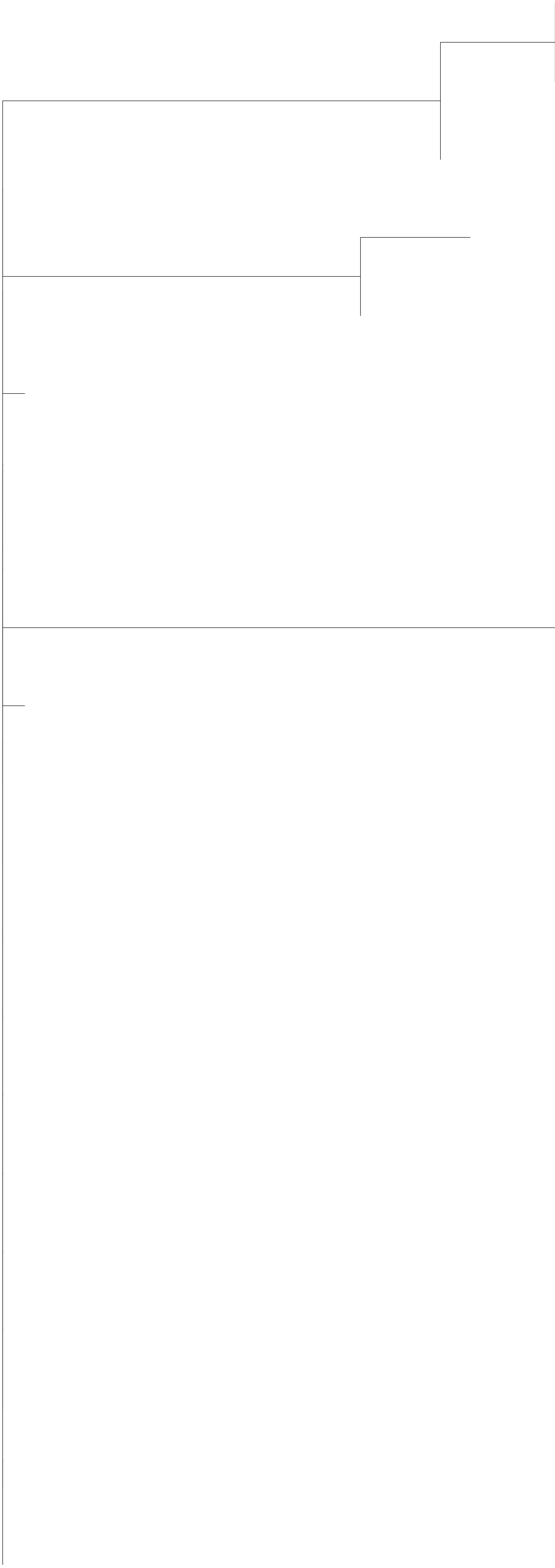





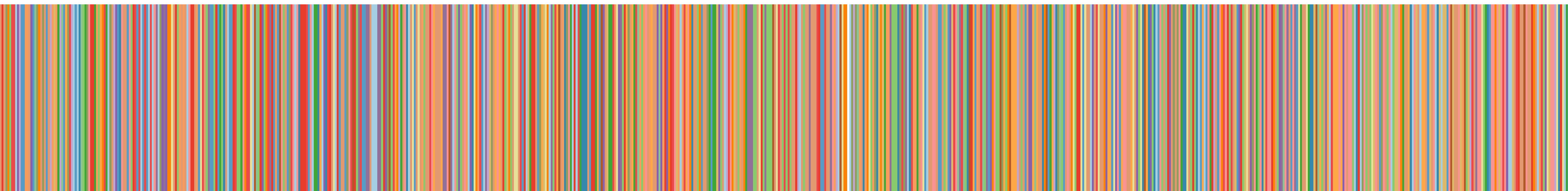



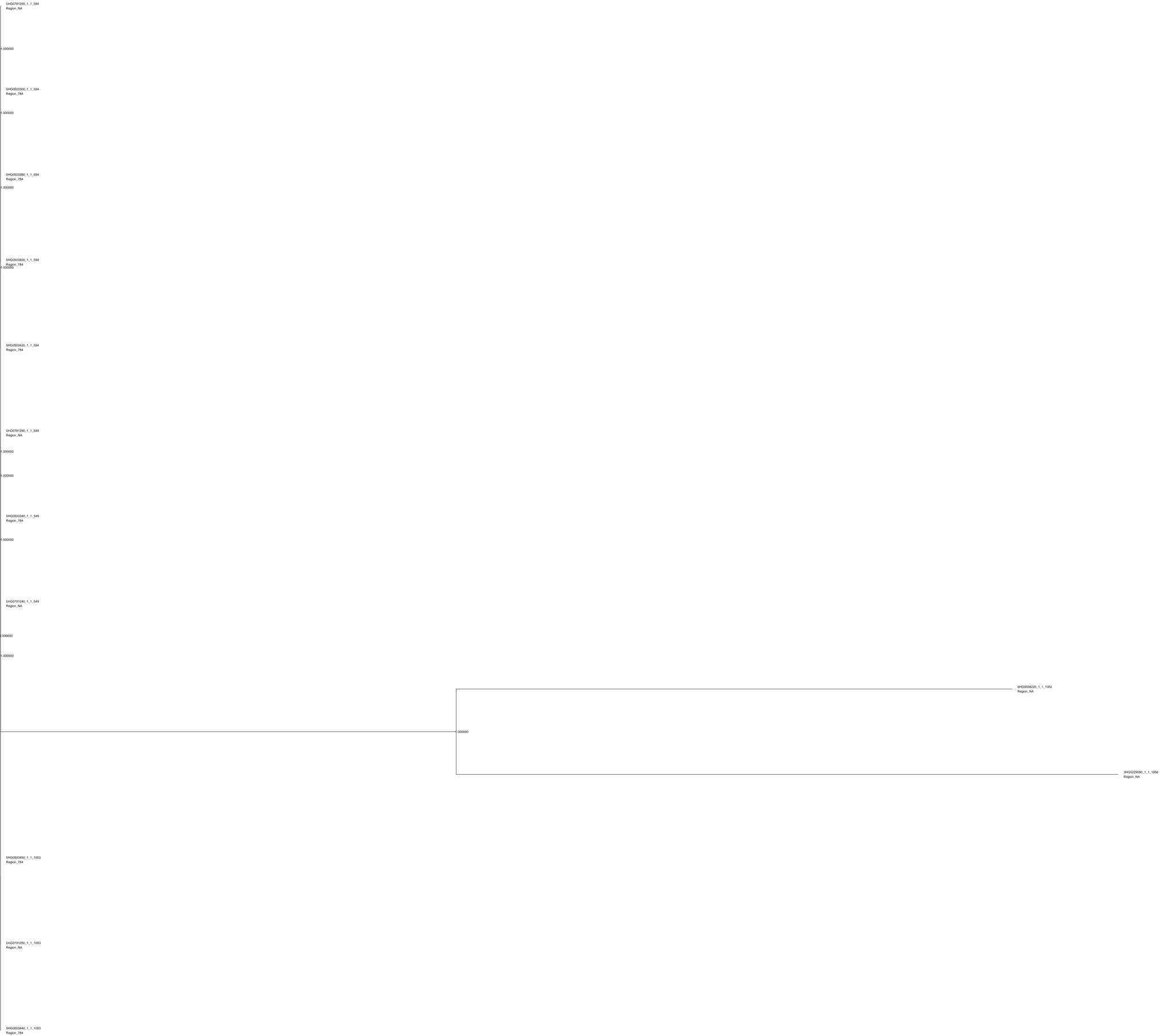











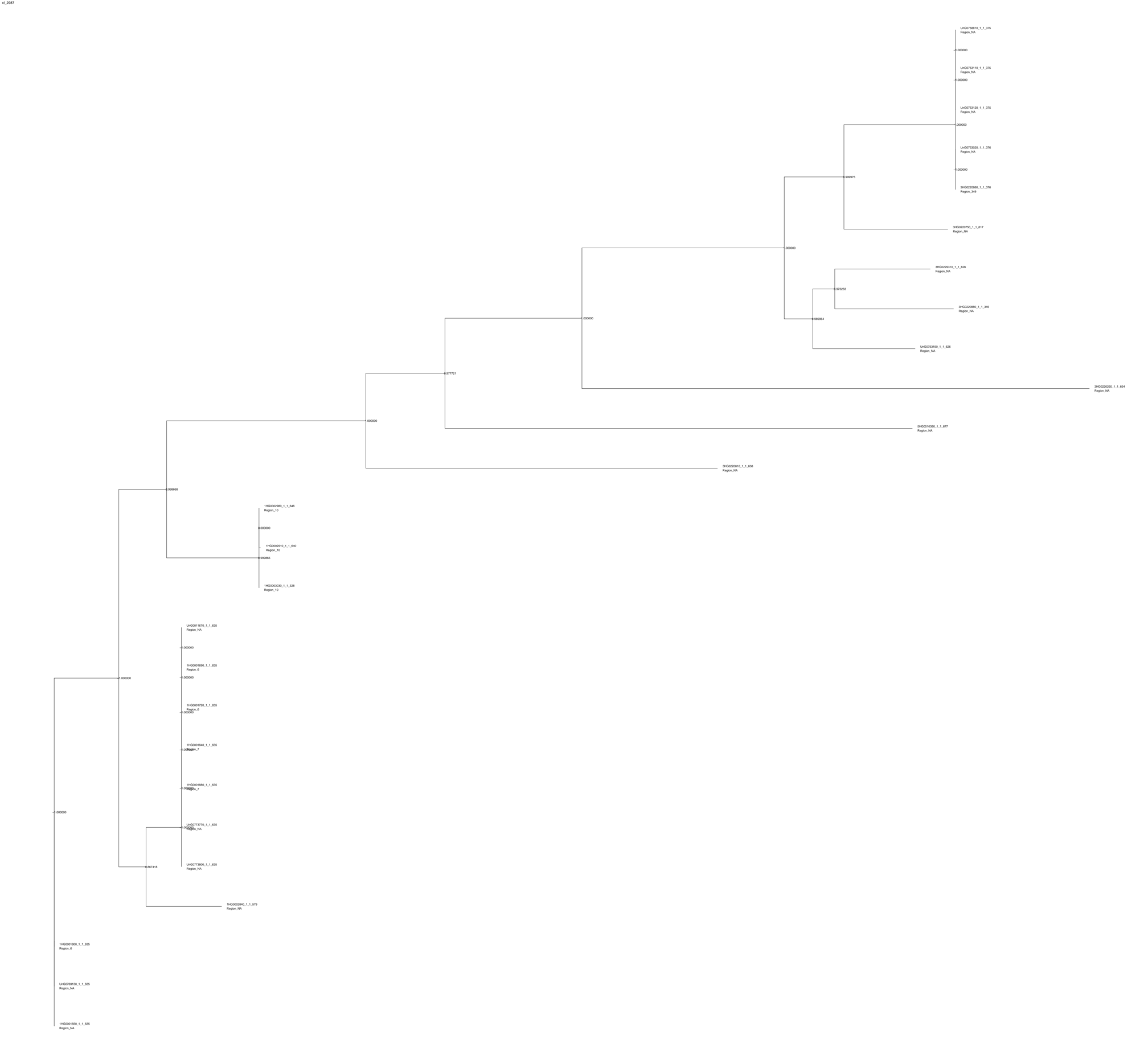

Supplement: Supplementary file 3 — Supplementary Material 3. [file 12870_2025_7328_MOESM3_ESM.zip › MTRW_RGS_SM_2.pdf]
